# A review on artificial intelligence for the diagnosis of fractures in facial trauma imaging

**DOI:** 10.3389/frai.2023.1278529

**Published:** 2024-01-05

**Authors:** Tuan D. Pham, Simon B. Holmes, Paul Coulthard

**Affiliations:** Barts and The London School of Medicine and Dentistry, Queen Mary University of London, London, United Kingdom

**Keywords:** facial trauma, facial-bone fractures, diagnosis, radiology, artificial intelligence

## Abstract

Patients with facial trauma may suffer from injuries such as broken bones, bleeding, swelling, bruising, lacerations, burns, and deformity in the face. Common causes of facial-bone fractures are the results of road accidents, violence, and sports injuries. Surgery is needed if the trauma patient would be deprived of normal functioning or subject to facial deformity based on findings from radiology. Although the image reading by radiologists is useful for evaluating suspected facial fractures, there are certain challenges in human-based diagnostics. Artificial intelligence (AI) is making a quantum leap in radiology, producing significant improvements of reports and workflows. Here, an updated literature review is presented on the impact of AI in facial trauma with a special reference to fracture detection in radiology. The purpose is to gain insights into the current development and demand for future research in facial trauma. This review also discusses limitations to be overcome and current important issues for investigation in order to make AI applications to the trauma more effective and realistic in practical settings. The publications selected for review were based on their clinical significance, journal metrics, and journal indexing.

## 1 Introduction

Fractures in facial trauma include damaged bones of the facial skeleton: forehead (frontal bone), zygoma (cheekbone), maxilla (upper jaw), mandible (lower jaw), nose, and orbit (eye socket). Oral and maxillofacial trauma describes soft and hard tissue injuries of the mouth and face. Trauma management has evolved significantly over the last few decades. There has been a focus on reducing mortality during the “golden hour” for patients experiencing polytrauma. There has also been an increasing emphasis on enquiring about the etiology of the presenting injury, in terms of safeguarding need, and understanding the nature of the physical injury. Facial fractures may result from interpersonal violence, road traffic accidents, falls, sports and industrial accident.

**Figure 1:**
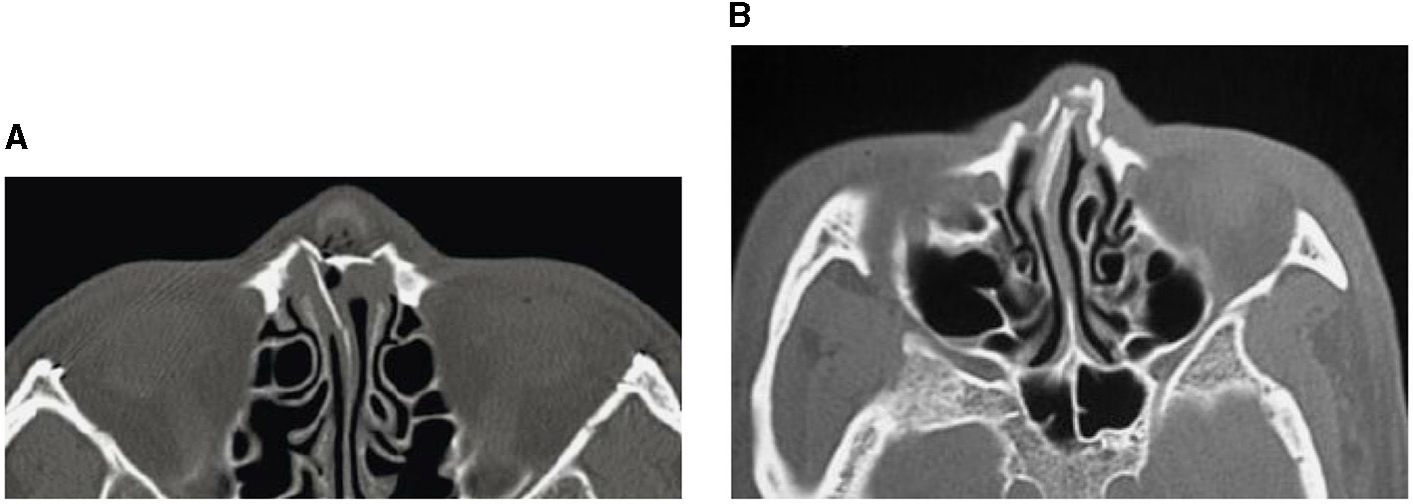
CT images showing nasal bone fractures: across the complete nasal bone **(A)**, and with leftward deviation **(B)**.

**Figure 2:**
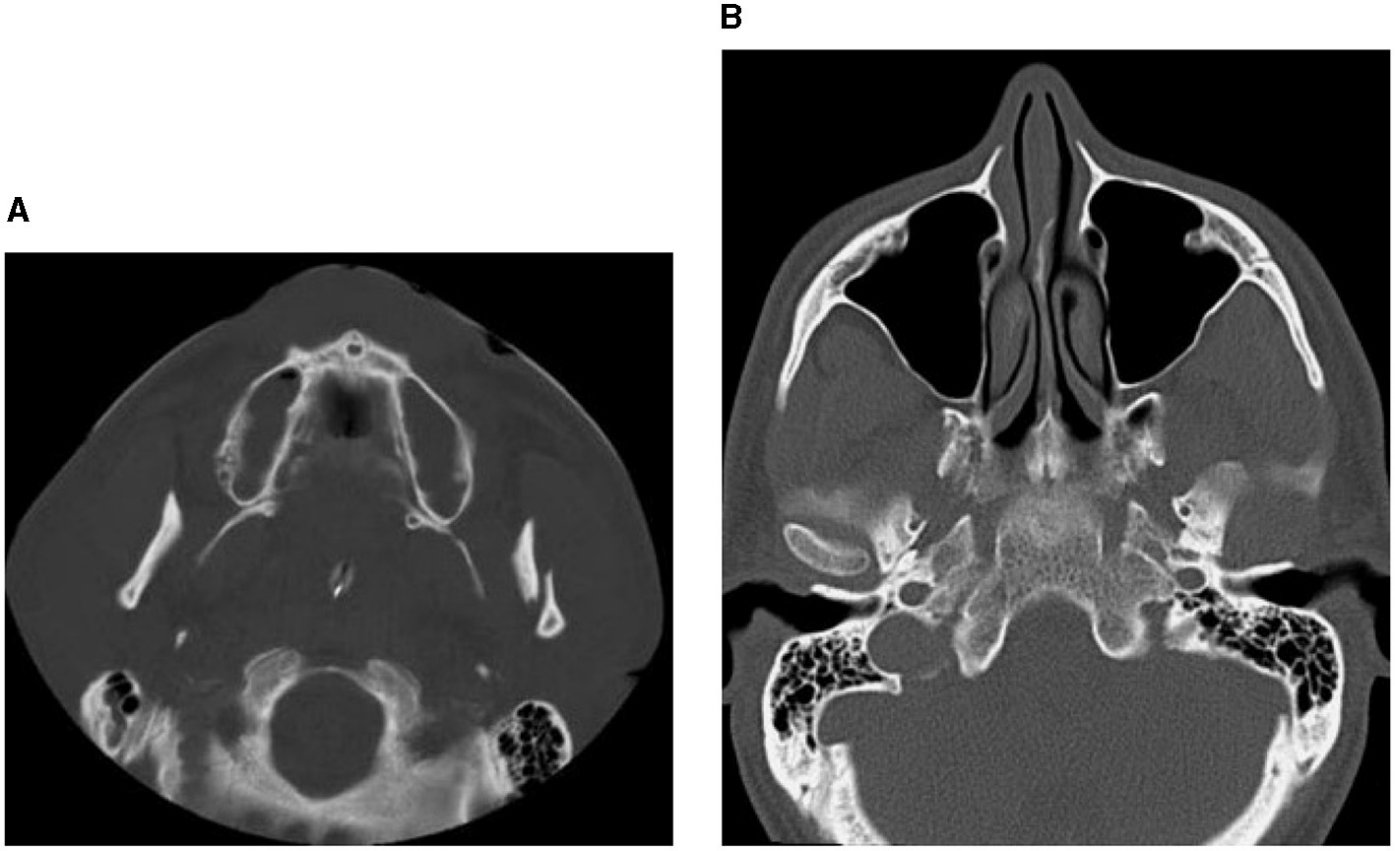
CT images showing factures of mandibular bones: a fracture of the left condyle, featuring lateral displacement of the proximal condylar fragment **(A)**, and condylar fracture with dislocation **(B)**.

**Figure 3:**
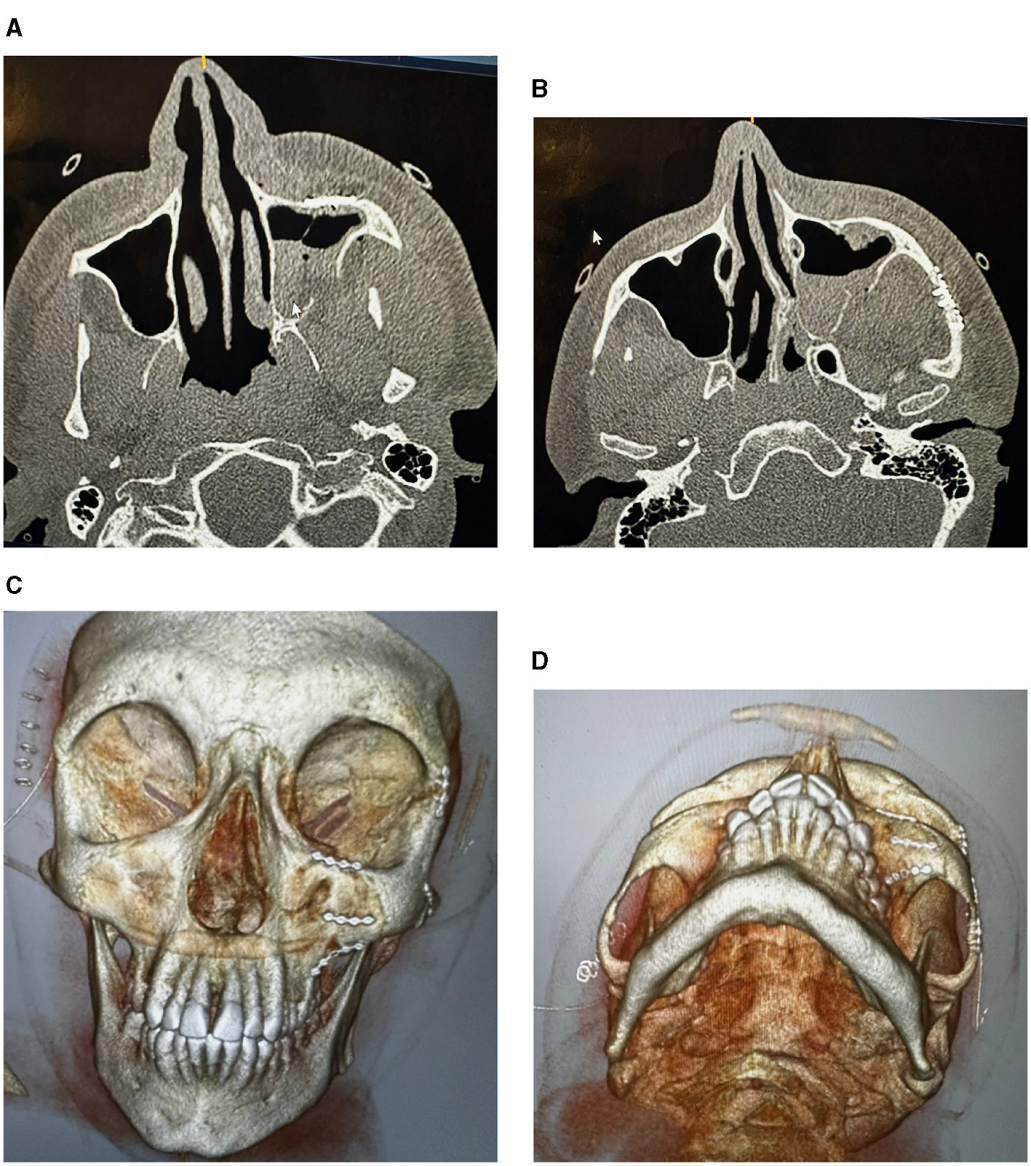
CT images showing factures of cheek and socket bones: before treatment **(A)**, and after reduction and fixation **(B–D)**.

Facial trauma is classified into injures of the lower, middle, and upper thirds of the face. The upper third includes the frontal bone, the mid-third includes the maxilla, zygomas, orbits, nose and naso-ethmoidal complex, whilst the lower third includes the mandible. Another classification of facial-bone injuries is midface fractures, which are also known as Le Fort fractures: Le Fort I, II, and III (Patel et al., 2022). This type of fractured bones is named after Rene Le Fort who studied the blunt force trauma of corpse skulls and defined the lines of weakness in the maxilla, where fractures occurred. Le Fort I is the fracture that extends above the maxilla; Le Fort II includes the lower area of one cheek across the bridge of the nose, and to the lower area of the other cheek; and Le Fort III is the fractured part that is across the nasal bridge and bones surrounding the eyes. The Le Fort classification was commonly adopted method for describing maxillary fractures. It is reserved to describe the precise occurrence according the initial description (Vujcich and Gebauer, 2018). Because of the complex structure of the facial bone framework, there are many other types of bones found in the deeper structure of the face (Facial Fractures, 2020). The structural, diagnostic, and therapeutic complexity of the individual midfacial subunits, including the nose, the naso-orbito-ethmoidal region, the internal orbits, the zygomaticomaxillary complex, and the maxillary occlusion-bearing segment are considered to be more important and of superior clinical relevance (Dreizin et al., 2018). Figures 1–3 shows some CT slides of facial bone fractures (nasal, mandible, orbit, and cheek).

Facial trauma accounts for a large number of admissions to the emergency department and a high level of morbidity and mortality (Hooper et al., 2019), mostly among the working-age population that are those whose ages are <40 years (Bocchialini and Castellani, 2019). Thus, facial trauma can result in the loss of productivity, which is greater than the population of patients with cancer or cardiomyopathy (Bocchialini and Castellani, 2019). Major facial bone fractures are caused by high-impact accidents such as traffic collisions, falls, sport injuries, and interpersonal violence. Fractures of the nasal bones (broken nose) are the most common type of facial trauma, which constitute 50% of cases (Atighechi and Karimi, 2009). Complex fractures of facial bones can suffer from irreversible impairments and may even be capable of causing death as a result of the airway obstruction or hemorrhage. Because of its proximity to the brain and central nervous system, facial trauma may cause damage to cranial nerves, result in losses of sensations, facial expressions, and eye movements (Mistry et al., 2023).

According to the updated information given by the Cleveland Clinic (Facial Fractures, 2020), facial fractures are treated by performing either a closed or open reduction. A close reduction is a non-surgical procedure for enabling repositioning of bony fragments and healing. An open reduction is a surgical procedure involving an incision for access to bony fragments, reduction, and fixation. For a complex facial trauma with multiple bony fractures, the performance of reconstructive surgery is needed. Particularly when a facial trauma is life-threatening, immediate treatment must be provided to avoid certain critical associated problems described earlier.

Accurate diagnosis of complex facial trauma, particularly midface injuries, is a challenge for radiologists with respect to understanding the anatomical classification (Rosello et al., 2020) and importance of fractures that are critical to inform surgeons for effective management of the trauma (Hopper et al., 2006). Discrepancies between reports provided by radiologists and surgeons due to different diagnoses are part of challenges for providing timely surgery to emergent cases of facial trauma (Ludi et al., 2016). To alleviate the problem of missing fractures from detection due to malocclusion, facial disfigurement, overlapping bony fragments, and obscure soft-tissue swelling, the use of 3D computed tomography (CT) imaging has been utilized in the diagnosis of maxillofacial fractures. However, surgical treatments were reportedly improved to only 33% of the cases (Shah et al., 2016). Furthermore, given the timing and order of surgeries in emergency departments, selecting optimal treatments for patients with complex facial fractures imposes a challenging task for radiologists, emergency physicians, and surgeons (Chung et al., 2013).

Although the Le Fort classification has been commonly used for diagnostics and treatment of facial fractures, it has certain critical shortcomings (Donat et al., 1998). The Le Fort fails to provide sufficient information, such as, for the fracture description, complete treatment plan, definitions of the facial skeletal supports and combined maxillary fractures, and description of fractured bones bearing the occlusal segment. The facial fracture complexity is also oversimplified by the Le Fort classification, which leads to the limited description of critical facial fracture patterns. CT is commonly used for assessing patients with blunt facial trauma. Advancement of medical scanners significantly improve the image resolution to allow the visualization of small fractures of the facial skeleton. However, in complex midface injuries, the ability to detect critical fractures that are needed to point out to trauma surgeons is still a challenge to radiologists (Hopper et al., 2006).

To aid physicians, radiologists, and surgeons in emergency departments, AI has been applied to assessing bone fractures in other fields of trauma medicine. Such recent applications include body-region fractures (shoulder and clavicle, spine, hand and wrist, elbow and arm, hip and pelvis, femurs, rib cage, foot and ankle, knee and leg, scaphoid, etc.) (Wu et al., 2013; Ozturk and Kutucu, 2017; Cheng et al., 2019, 2021; Bluthgen et al., 2020; Jones et al., 2020; Krogue et al., 2020; Ajmera et al., 2021; Duron et al., 2021; Lind et al., 2021; Ren and Yi, 2021; Sato et al., 2021; Yoon et al., 2021; Guermazi et al., 2022; Liu et al., 2022, 2023; Murphy et al., 2022; Nguyen et al., 2022; Oakden-Rayner et al., 2022; Bousson et al., 2023; Gao et al., 2023; Hendrix et al., 2023). Reviews of AI applications for detecting non-facial bone fractures in adults and children have been reported in the literature (Lindsey et al., 2018; Kalmet et al., 2020; Rainey et al., 2021; Cha et al., 2022; Dankelman et al., 2022; Kuo et al., 2022; Meena and Roy, 2022; Shelmerdine et al., 2022; Zech et al., 2022).

This paper aims to contribute an updated critical review on applications of AI to the treatment and management of facial trauma with a special reference to the image-based prediction/classification of bone fractures. The rest of this paper is organized as follows. As a background for the review on AI applications to the image analysis and prediction of fractured bones in facial trauma, different image modalities used for the diagnostics will be presented. Subsequent sections are the review of how AI systems can learn for automated image analysis and detection of facial fractures, discussions of current research and outline of next steps for investigation, and concluding remarks of the review.

## 2 Inclusion and exclusion criteria

In the context of this literature review on AI applications for diagnosing fractures in facial trauma imaging, the utilization of inclusion and exclusion criteria plays a crucial role in delineating the scope of the review. These well-defined criteria serve the purpose of incorporating pertinent and high-quality research while concurrently excluding studies that might deviate from the intended objectives. Specific inclusion and exclusion criteria applied in this literature review are outlined as follows.

### 2.1 Inclusion criteria

- Publication type: include peer-reviewed journal articles, conference proceedings, and reputable research reports.
- Publication date: include articles published from 2010 to the present to focus on recent advancements in AI for facial trauma diagnosis.
- Language: include articles published in the English language.
- Study focus: include studies that specifically address AI applications for the diagnosis of facial fractures. This includes studies discussing AI algorithms, techniques, or tools developed for fracture detection, classification, or assessment in facial trauma imaging. Because of limited work on AI applications for image-based fracture diagnosis in facial trauma, a related topic on skull fracture detection is included in this review.
- Study design: include various study designs, such as experimental studies, clinical trials, retrospective analyses, and case studies, as long as they contribute to the understanding of AI applications in diagnosing facial fractures.
- Population: include studies involving human patients with facial trauma or relevant imaging data.
- Interventions: include studies that describe or assess AI-based interventions, including machine learning algorithms, deep learning models, or computer-aided diagnosis systems, applied to facial trauma imaging.
- Outcome measures: include studies reporting outcomes related to AI performance, accuracy, sensitivity, specificity, or any relevant diagnostic metrics for facial fracture detection.

### 2.2 Exclusion criteria

- Publication type: exclude non-academic sources, opinion pieces, and editorials.
- Publication date: exclude studies published before 2010 unless they are seminal works or provide crucial historical context.
- Language: exclude studies published in languages other than English.
- Study focus: exclude studies that do not address AI applications in facial fracture diagnosis or those that focus on unrelated medical conditions.
- Study design: exclude studies with methodological flaws, limited sample sizes, or poor experimental design that could compromise the validity of their findings.
- Population: exclude studies that do not involve human patients with facial trauma or relevant imaging data.
- Interventions: exclude studies that do not involve AI-based interventions for facial fracture diagnosis.
- Outcome measures: exclude studies that do not report relevant outcomes related to ai performance or diagnostic accuracy.
- Duplications: exclude duplicate publications or multiple reports of the same study to avoid redundancy.

## 3 Conventional machine learning and deep learning

Artificial intelligence (AI) and its subsets known as machine learning and particularly the methods of deep learning (Esteva et al., 2019) have been reportedly providing effective solutions to many complex problems in health and medicine in timely and efficient ways.

In conventional machine learning for pattern classification, a selected classifier is trained with features extracted from the training data by a manually selected algorithm for feature extraction. In deep learning, such as a convolutional neural network (CNN), features are extracted through many hidden layers from the training images by the network itself. The depth of a CNN architecture, which can be up to about-hundred layers, is so called deep learning. A CNN has two main parts: deep feature extraction and deep feature learning for classification. The deep feature extraction involves repeated executions of three operations known as convolution, nonlinearity, and pooling (downsampling). The learning phase for classification involves the vectorization or flattering the extracted deep features, shared weights and biases, and classification layers. The layer being next to the last layer of the network is a fully connected layer that produces a vector of probabilities for the number of image classes that the network has been trained on. Unlike a traditional (shallow) neural network, the shared weights and bias values of a CNN, which are the same for all hidden artificial neurons (nodes) in a given layer. This design makes a CNN robust to translational orientations of patterns by being able to detect an object wherever it is present in the image.

**Figure 4:**
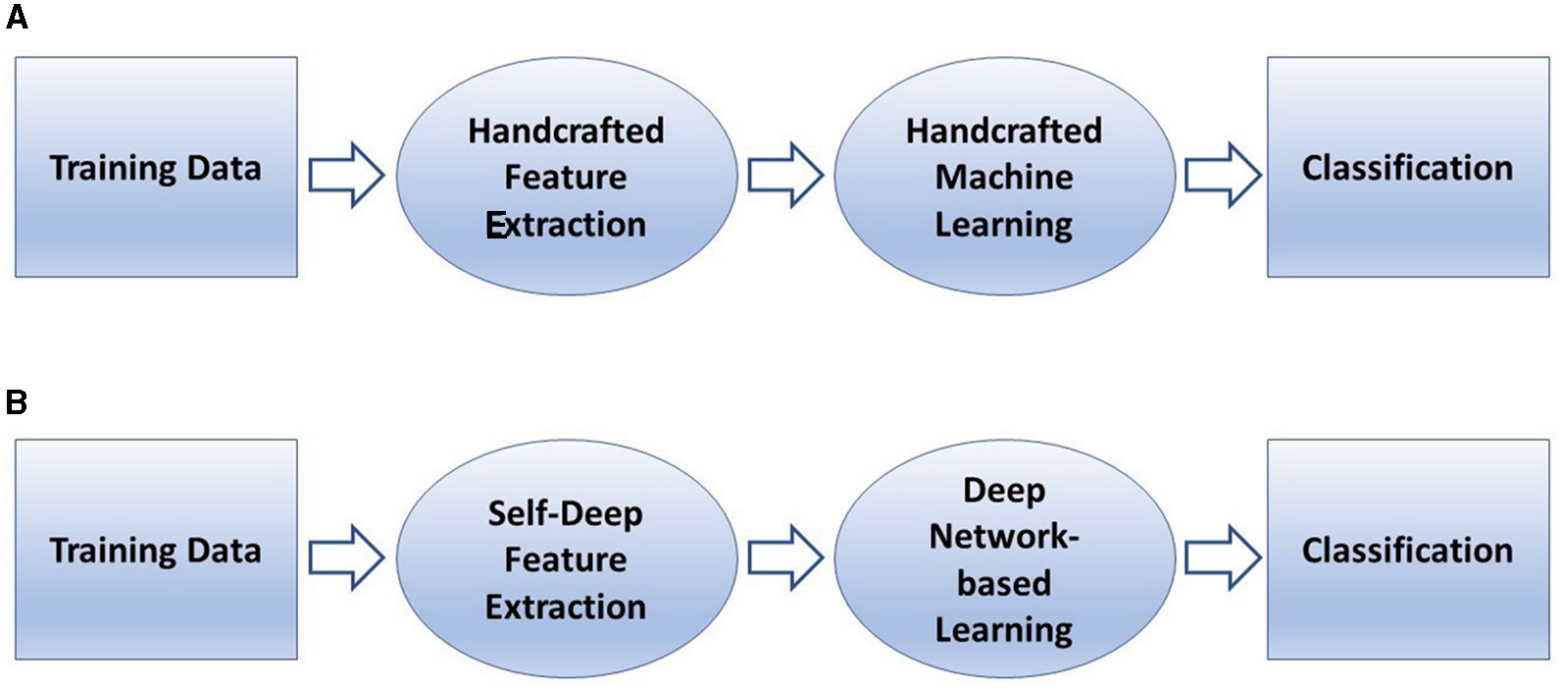
Learning with AI: conventional machine learning **(A)**, and deep learning **(B)**.

**Figure 5:**
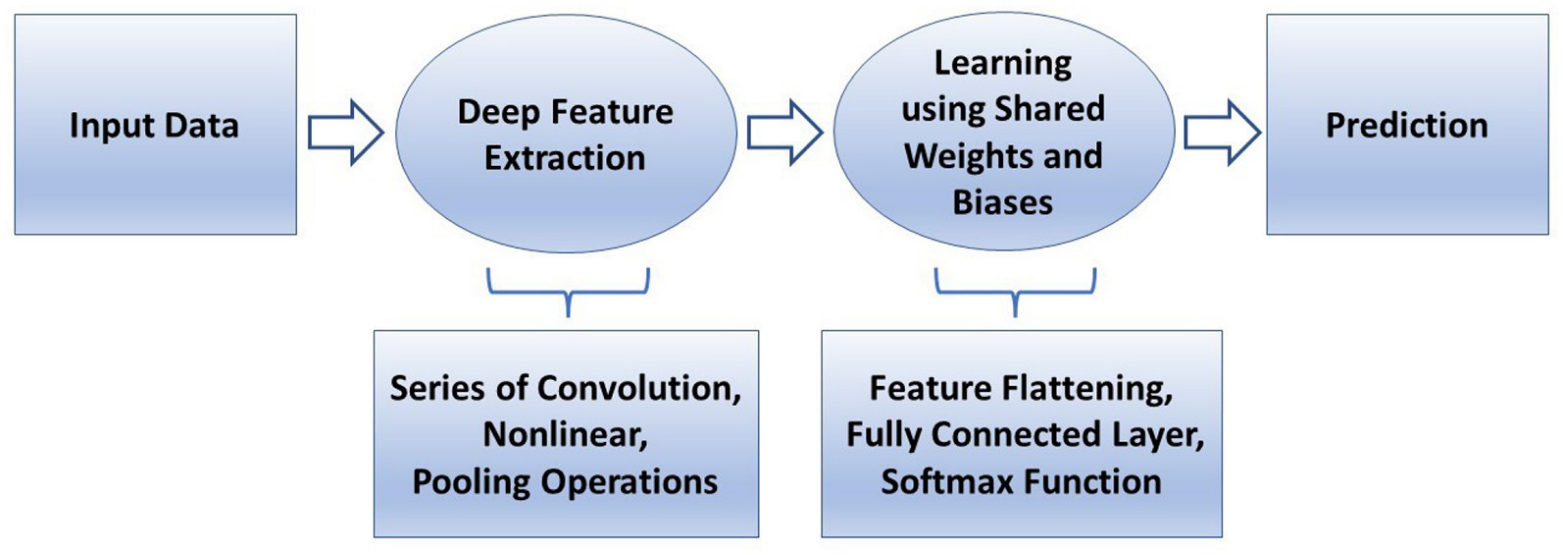
Semantic architecture of a convolutional neural network.

Figure 4 describes a basic comparison between conventional machine learning and deep learning procedures, and Figure 5 shows the semantic architecture of a CNN.

Furthermore, generative models and classification models are two fundamental paradigms in machine learning, each serving distinct purposes in data analysis and decision-making. Generative models, such as variational autoencoders (VAEs) and generative adversarial networks (GANs), focus on learning the underlying data distribution and generating new data points that resemble the training data. They are particularly useful for tasks like image synthesis, text generation, and data augmentation. In contrast, classification models, like support vector machines (SVMs) or convolutional neural networks (CNNs), are designed for the task of assigning input data to predefined categories or classes. They excel in tasks such as image recognition, natural language processing, and sentiment analysis. While generative models enable creativity and data generation, classification models empower decision-making and pattern recognition, collectively contributing to the diverse landscape of machine learning applications.

As a review paper on AI for facial fracture diagnosis, detailed equations and figures for CNN architectures are not addressed for some reasons:

- Scope of the review: review papers typically aim to provide a comprehensive overview of existing literature, methodologies, and trends in a specific field. While CNNs are important in AI for image analysis, including detailed equations and figures for a specific architecture may be beyond the scope of the review, which should focus on broader trends, challenges, and applications.
- Variability in architectures: CNN architectures can vary significantly depending on the specific task, dataset, and research objectives. Providing equations and figures for one or few some particular CNN architectures may not be representative of the diverse approaches used in facial fracture diagnosis.
- Rapid evolution: the field of AI, including CNNs, is constantly evolving with new architectures, techniques, and innovations emerging regularly. Detailed equations and figures for a specific architecture may quickly become outdated. It is more practical to discuss general principles and trends that are likely to persist over time.

The foundational principles and current trends in the field of CNNs, particularly in the context of the applications discussed in this review, encompass the following aspects:

- Hierarchical feature extraction: CNNs are fundamentally built on the idea of hierarchical feature extraction. They consist of multiple layers, including convolutional layers and pooling layers, that progressively learn and extract features from input data. This principle of hierarchical abstraction is likely to remain at the core of CNN design.
- Convolution and filters: the convolution operation, which involves sliding filters over input data to detect patterns and features, is a foundational concept in CNNs. While filter designs may evolve, the concept of local feature detection through convolution is expected to persist.
- Depth and stacking: deeper networks have consistently shown improved performance in various computer vision tasks. This trend toward deeper architectures is likely to continue, although optimization techniques like skip connections and residual networks may mitigate some of the challenges associated with depth.
- Transfer learning: transfer learning, where pre-trained models on large datasets are fine-tuned for specific tasks, is a powerful trend in CNNs. It enables the application of models trained on general image recognition tasks to medical image analysis, including facial fracture diagnosis.
- Data augmentation: data augmentation techniques, such as rotation, scaling, and flipping, are used to increase the effective size of training datasets. These techniques help CNNs generalize better and are expected to remain crucial.
- Interpretability: interpretability and explainability of CNNs are growing concerns, especially in medical applications. Future trends may include the development of CNN architectures that provide more transparent decision-making processes, which is vital for medical professionals.
- Efficiency and hardware acceleration: as CNNs become more complex, there's a need for efficient implementations. Trends will likely focus on optimizing models for deployment on specialized hardware like GPUs, TPUs, or even neuromorphic chips.
- Regularization techniques: techniques like dropout, batch normalization, and weight decay are used to prevent overfitting. They are expected to remain relevant as researchers seek to make CNNs more robust and generalizable.
- Integration with clinical workflow: future trends may involve the integration of CNN-based systems into clinical workflows. This includes ensuring that AI systems meet regulatory and ethical standards while providing actionable insights to healthcare professionals.
- Ethical considerations: as AI becomes more prevalent in medical diagnosis, ethical considerations such as patient privacy, bias mitigation, and accountability will continue to be important trends to address.

## 4 Image modalities for the diagnostics of facial trauma

If a fracture is suspected, an emergency or trauma physician may order an initial imaging examination to determine the location and the fracture type. Because there are several types of fractures, there are different imaging modalities for suitable examination of the suspected injury. A variety of diagnostic imaging methods are recommended for evaluating facial trauma based on the following locations of the injury (Expert Panel on Neurological Imaging et al., 2022):

1. Forehead or frontal bone
2. Midface
3. Nose
4. Mandible

Conventional radiography has historically played a significant role in the diagnosis of maxillofacial fractures. Nevertheless, its utility is limited when it comes to visualizing overlapping bones, soft-tissue swelling, and the dislocation of facial fractures (Shah et al., 2016). To address these limitations, computed tomography (CT) has emerged as the prevailing diagnostic imaging modality. The American College of Radiology recommends specific initial imaging procedures for evaluating facial trauma following a primary survey aimed at rapidly assessing and treating life-threatening injuries, as outlined in Table 1. It is worth noting that while magnetic resonance imaging (MRI) and magnetic resonance angiography (MRA) are included in this list, they are typically not the primary modalities used for fracture detection, particularly in acute settings.

**Table 1:** Approptiate initial imaging evaluation of facial trauma following primary survey (Expert Panel on Neurological Imaging et al., 2022).

**Trauma location**	**Imaging procedure**
Forehead	CT maxillofacial without intravenous contrast
	CT head without intravenous contrast
Midface	CT maxillofacial without intravenous contrast
Nose	CT maxillofacial without intravenous contrast
	US maxillofacial
	Radiography paranasal sinuses
Mandible	CT maxillofacial without intravenous contrast
	Radiography mandible

Accurate diagnosis of fractures in patients with facial trauma is a critical aspect of medical care, and various imaging modalities play a pivotal role in achieving precise assessments and treatment planning. In this context, advanced imaging techniques offer valuable insights into the extent and nature of the fractures, enabling clinicians to formulate tailored treatment strategies that address both the immediate and long-term healthcare needs of the patients. Additionally, the integration of cutting-edge technologies, including AI models, has further augmented the capabilities of medical practitioners in accurately identifying and characterizing complex fracture patterns, thereby facilitating timely interventions and improving patient outcomes. Such advancements not only enhance the diagnostic accuracy but also contribute to the optimization of surgical procedures, ultimately ensuring comprehensive and patient-centric management of facial trauma injuries.

One of the most frequently employed imaging modalities for this purpose is X-ray radiography, which furnishes 2D images of the facial bones. X-rays excel at swiftly identifying fractures, gauging their severity, and determining the alignment of fractured bone fragments. They prove particularly valuable for detecting fractures in areas like the mandible, maxilla, and nasal bones. Furthermore, X-ray imaging boasts the advantages of being non-invasive, readily accessible, and cost-effective, rendering it a primary choice in many emergency departments for the initial evaluation of facial trauma.

Having mentioned earlier, another indispensable imaging modality in the realm of diagnosing facial fractures is CT. CT scans, including cone beam CT (CBCT), provide high-resolution 3D images of the facial bones, supplying intricate details about the extent and complexity of fractures. This modality is especially beneficial when assessing fractures of the orbital bones, zygoma, and complex mid-face injuries. CT scans facilitate precise surgical planning and are indispensable for evaluating potential complications, such as airway compromise, vascular injury, or brain involvement in severe facial trauma cases. The capacity to visualize fractures from multiple angles and with exceptional detail makes CT an indispensable cornerstone in the diagnostic workup of facial fractures.

Different imaging modalities come with various levels of radiation exposure and associated risks. X-rays are a form of ionizing radiation that can potentially pose health risks, especially with repeated exposure. Overexposure to X-rays can increase the risk of tissue damage, cellular mutations, and potentially cancer development. To minimize the risks, radiation doses should be kept as low as reasonably achievable. Modern X-ray systems are designed to reduce unnecessary radiation exposure while maintaining image quality.

CT scans utilize X-rays in a more sophisticated manner, resulting in higher radiation exposure compared to conventional X-ray imaging. Repeated or high-dose CT scans can significantly increase the risk of radiation-related complications, such as cancer, especially in younger patients. Dose reduction techniques, such as lowering tube current, using appropriate scanning protocols, and employing iterative reconstruction algorithms, should be implemented to minimize radiation exposure while maintaining image quality.

CBCT employs a cone-shaped X-ray beam, resulting in higher radiation exposure compared to traditional dental X-rays. Prolonged or repeated exposure to CBCT imaging can potentially lead to an increased risk of radiation-related complications, particularly in sensitive areas of the head and neck. Being similar to conventional CT scans, implementing dose reduction strategies is crucial in minimizing radiation exposure during CBCT imaging. The necessity of CBCT scans and alternative imaging methods should be considered, especially for cases where lower radiation exposure options are available.

Balancing the diagnostic benefits of these imaging modalities with their associated risks is crucial in clinical practice. Implementing appropriate protocols and using radiation protection measures can help mitigate the potential repercussions of radiation exposure during medical imaging procedures.

## 5 AI for fracture detection in facial trauma

Below are reviews of various AI methods that have been developed and employed for the detection of fractures in facial trauma cases. This review paper offers insights into the diverse applications of AI techniques in identifying and characterizing complex fracture patterns within medical imaging data. While the scope of this paper focuses primarily on discussing the performance and efficacy of these AI methods, it is essential to acknowledge the significance of detailed image dataset characteristics, which contribute to the robustness and reliability of the proposed fracture detection models. Readers seeking in-depth knowledge about the specific attributes and compositions of these image datasets are encouraged to refer to the relevant references provided within this review paper. By consulting these referenced sources, readers can gain a more comprehensive understanding of the intricate details associated with the image datasets, ensuring a holistic perspective on the methodologies and outcomes discussed in the context of fracture detection in facial trauma. This approach not only maintains the focus on the key findings and implications presented in the review but also fosters an appreciation of the underlying data-driven insights and advancements in this critical area of medical imaging research.

### 5.1 Frontal-bone fractures

Up to date, it seems that AI has not been found its application to predicting fractures of the frontal bone in facial trauma. Similar studies were found for AI-based detection of critical information of head trauma that needs urgent attention. Using non-contrast head CT scans, Chilamkurthy et al. (2018) applied deep-learning methods [ConvNets (Simonyan and Zisserman, 2014)] for detecting calvarial fractures, midline shift, mass effect, and five types of intracranial hemorrhage, which are intraparenchymal, intraventricular, subdural, extradural, and subarachnoid. The authors collected more than 313,000 head CT scans from around 20 centers in India for training and validating the deep learning. Although the results showed the potential application of the deep learning for detecting critical abnormalities on head CT scans, it seemed that the authors randomly selected a subset of the same training data for validation, which was not supposed to be used, in addition to a another validation dataset. Furthermore, the technical development of the deep learning approach was not well described as pointed out by other authors (Liu et al., 2019).

Wang et al. (2023) presented a deep learning framework designed to automatically identify bone fractures in both cranial and facial regions. Their system integrates YOLOv4 for streamlined fracture detection and ResUNet++ (Jha et al., 2019) for segmentation of cranial and facial bones. By consolidating the outcomes of both models, the authors could pinpoint the fracture location and identified the affected bone. The training dataset for the detection model comprised soft tissue algorithm images extracted from a comprehensive set of 1,447 head CT studies (comprising 16,985 images), while the segmentation model was trained on a carefully selected 1,538 head CT images. The models underwent testing on a separate dataset of 192 head CT studies (5,890 images). The approach achieved a sensitivity of 89%, precision of 95%, and an F1 score of 0.91. Specifically, evaluation of the cranial and facial regions demonstrated sensitivities of 85% and 81%, precisions of 93% and 87%, and F1 scores of 0.89 and 0.84, respectively. The average accuracy for segmentation labels, considering all predicted fracture bounding boxes, was 81%.

### 5.2 Midfacial fractures

Ryu et al. (2021) carried out a literature survey on the applications of AI methods to other fields of medical imaging diagnostics that are potential aiding craniofacial surgery, including craniofacial trauma, congenital anomalies, and cosmetic surgery. The survey particularly discussed the potential use of CNN, recurrent neural network (RNN), and generative adversarial network (GAN) models. These AI methods belong to the family of deep learning. These authors suggested it is important for craniofacial surgeons to understand the concepts and capabilities of different deep-learning methods in order to know how to appropriately utilize them and evaluate their performance.

Warin et al. (2023) evaluated the performance of CNN models for the CT-based detection and classification of maxillofacial fractures. The authors used 3,407 CT images, in which 2,407 images contained maxillofacial fractures and other 1,000 maxillofacial CT images were without fracture lines or pathologic lesions. The imaging data were obtained from a regional trauma center. Maxillofacial fracture lines on CT images were examined and annotated as the ground truth by the consensus of five oral and maxillofacial surgeons who had more than 5 years of experience in maxillofacial trauma. The detection involved a multi-class image classification task to categorize maxillofacial fractures into frontal, midface, mandibular and no fracture classes. The adopted CNN models were DenseNet-169 and ResNet-152 for image classification, and Faster region-based CNN (Faster R-CNN) (Ren et al., 2017) and YOLOv5 (Jocher et al., 2022) for object detection in images. The frontal, midface and mandibular fracture images were randomly split into training, validation, and test sets using the ratio of 70:10:20%, respectively. DenseNet-169 achieved the best overall accuracy of the best multiclass classification (70%), while Faster R-CNN provided the best average precision (78%). Limitations of the study pointed out by the authors include (1) the lack of data obtained from multiple centers (data from only two centers were used) that could not ensure the robustness and generalization of the AI models; (2) low image resolution (512 × 512 pixels) that adversely affected the model accuracy; and (3) the lack of other image views such as coronal and sagittal plans for enabling the detection of local sites of maxillofacial fractures.

Amodeo et al. (2021) adopted ResNet50, which is a pretrained CNN model, for maxillofacial fracture detection using CT scans. The number of CT scans corresponded to the number of patients, where a CT scan was obtained for each patient. The total dataset consisted of 208 CT scans of 208 patients, of which 11,260 image slices were of the fracture class, and 49,762 image slices belonging to the class of non-fracturing. The data were split into training (120 scans of fracture and 28 images of non-fracturing), validation (25 images of fracture and five images of non-fracturing), and test sets (25 images of fracture and five images of non-fracturing). A diagnosis was determined as having a fracture if two consecutive slices were classified with the probability of fracture > 0.99. The test results showed a binary classification accuracy of 80%. Shortcomings of the AI model for fracture detection as outlined by the authors are that it was not able to detect tiny fractures and corner-bone fractures. More or less this study was a proof of concept for AI-based maxillofacial fracture detection on CI imaging, with more technical developments and inclusion of data variety to entail.

### 5.3 Nasal fractures

Yang et al. (2022) tried to compare the performance of detecting nasal-bone fractures between AI-based and radiologist-based readings of CT imaging. The data consisted of enrolled 252 patients. The AI method was a deep-learning model. The human team comprised 20 radiologists with different levels of experience. Two radiologists were tasked to determine the radiologic findings. It was reported that the AI-based detection system achieved higher sensitivity (94%), specificity (90%) than the radiologists' readings of the CT images. However, there was no improvement in the fracture detection of the AI model over the radiologists who had between 11 and 15 years of reading experience.

Seol et al. (2022) investigated the use of deep learning for 3D-imaging diagnosis of nasal fractures. Facial CT data were obtained from 2,535 patients, where there were 1,350 images of normal nasal bones and 1,185 images of fractured nasal bones. The study included only CT images that were scanned <2 days after fracture onset to avoid the effect of bone deformation after fracture. The AI models were 3D-ResNet34 and 3D-ResNet50. The CT images were reconstructed to isotropic voxel data with the whole region of the nasal bone represented in a fixed cubic volume. The data were split into training (864 of normal condition and 758 of fracture), validation (216 of normal condition and 190 of fracture), and test (270 of normal condition and 237 of fracture) sets for the training and evaluation of the deep-learning (AI) models. Binary classification results obtained from the five-fold cross-validation showed the areas under the receiver operating characteristic curve (AUC) of 0.95 and 0.93 provided by 3D-ResNet34 and 3D-ResNet50, respectively. A limitation of the learning capacity of 3D-ResNet34 and 3D-ResNet50 was that they likely failed to recognize fractures with various deformed shapes. The authors reported that out of about 150 cases, both deep networks produced detection errors in several cases, where the cubic voxel data consisted of minor fractures without depressed fractures or deviated nose. The two AI-based models also misclassified small fractures when their shapes were similar to those of normal nose categories.

Moon et al. (2022) adopted the YOLOX, which is a deep-learning-based image object detector, for classifying different types of facial bone fractures. The authors used 65,205 facial bone CT images obtained from 690 patients, of which about 5,000 bounding boxes of nasal bone fractures were extracted for machine learning (training). For constructing validation data, 4,681 facial bone CT images obtained from 50 patients, of which about 500 bounding boxes of nasal bone fractures were extracted. The test data consisted of about 400 bounding boxes extracted from CT images of 20 patients with nasal bone fractures and 20 patients without having fractures. It was pointed out that the AI model was trained with only image boxes of nasal bone fractures, it was tested against data that comprised facial bone fractures other than nasal fractured bones. The purpose was to investigate if the AI detector could recognize unforeseen non-nasal bone fractures of patients with facial trauma. The positive class was defined as the class of fracture, and the negative class indicated there was no fracture in the bounding box. The task of the trained deep network was to predict if each bounding box was either true positive (TP), false positive (FP), or false negative (FN). Taking TP, FP, and FN into account, the AI model obtained an average precision of 70% and a sensitivity/person of 100%. Facing a similar difficulty encountered by other studies reviewed earlier, the authors reported that the detection of small objects of fractures was a difficult task for the AI model. Furthermore, the ability of the deep learning to detect facial-bone fractures from different image views was also not considered in the study.

### 5.4 Mandibular fractures

Vinayahalingam et al. (2022) investigated the automated detection of mandibular fractures on panoramic radiographs (PRs) with the use of Faster R-CNN, where the Swin-Transformer (Liu et al., 2021) was implemented as a backbone of the network. The data consisted of 6,404 PRs obtained from 5,621 patients, in which 1,624 PRs were with fractures and 4,780 PRs were without fractures. Mandibular fractures on PRs were classified and annotated with bounding boxes based on electronic medical records. The bounding boxes fully enclosed the fracture lines. Regarding dislocated fractures, the bounding boxes included both fracture margins and gaps. The annotations were reviewed and revised by the consensus of three clinicians, who had at least five years of clinical experience. The training data consisted of 364 angle fractures, 492 condyle fractures, 61 coronoid fractures, 187 median fractures, 487 paramedian fractures, and 180 ramal fractures. The validation data consisted of 44 angle fractures, 61 condyle fractures, eight coronoid fractures, 23 median fractures, 58 paramedian fractures, and 22 ramal fractures. The test data consisted of 45 angle fractures, 65 condyle fractures, seven coronoid fractures, 24 median fractures, 60 paramedian fractures, and 21 ramal fractures. The AI method achieved an AUC = 0.98 and F1-score (combination of precision and recall scores) = 0.95. Some limitations of the study being pointed out by the authors include the lack of data obtained multiple centers to ensure the generalization of the adopted model, the lack of device variety for acquiring the PRs to validate its classification robustness as the data used were obtained with only two different scanners, and the trained AI system was not tested with data in clinical settings.

Wang et al. (2022) used two pretrained CNNs, which are the U-Net and ResNet, for detecting and classifying nine subregions of mandibular fractures on spiral CT scans. The CT data, which were obtained from 686 patients with mandibular fractures, were classified and annotated by three experienced maxillofacial surgeons. There were 222, 56, and 408 CT scans used for the training, validating, and testing the deep networks, respectively. The AI models achieved classification accuracies >90%, and mean AUC = 0.96.

Because the diagnosis of mandibular fractures on panoramic radiographic (PR) images is quite difficult, CBCT imaging is preferred by radiologists and surgeons. To alleviate the unavailability of CBCT, Son et al. (2022) combined two deep networks U-Net and YOLOv4 for detecting the location of mandibular fractures based on PR images without the use of CBCT. Another motivation of for the combination of the two deep networks was to minimize the number of likely undiagnosed fractures (symphysis, body, angle, ramus, condyle, and coronoid regions). The U-Net was effective for performing the semantic segmentation of mandibular fractures spreading over a wide area, it could better detect fracture lines in the symphysis, body, angle, and ramus regions. The YOLOv4 was complementary to the U-Net by providing better detection of fractures in the condyle and coronoid in the upper region of the mandible. The training data consisted of 360 PRs of mandibular fractures. The test data consisted of 60 PRs of mandibular fractures. The task was to classify a mandibular fracture into six anatomical types: (1) symphysis, (2) body, (3) angle, (4) ramus, (5) condylar process, and (6) coronoid process. The first three types are of middle fractures and the last two types are of side fractures. The combined approach resulted in the precision of 95%, recall of 87%. This study demonstrated the complimentary combination of U-Net and YOLOv4 networks that could improve the detection of mandible fractures by more than 90% (measured in terms of F1-score) than the individual networks.

### 5.5 Fractures in close proximity

A particular type of fracture that occurs in close proximity to facial bone fractures is known as a fractured skull bone. A skull fracture entails a break in the cranial bone structure and can be categorized into four main types as follows (Head Injury, 2023).

Linear skull fractures: linear fractures are the most common type of skull fractures. They involve a break in the bone but do not displace it. Patients with linear fractures are typically observed in the hospital for a short period and can often resume normal activities within a few days. Generally, no further medical interventions are necessary.

Depressed skull fractures: this type of fracture can be observed with or without a scalp laceration. In a depressed skull fracture, a portion of the skull is indented due to the trauma. The severity of this type of skull fracture may necessitate surgical intervention to correct the deformity.

Diastatic skull fractures: diastatic fractures occur along the suture lines in the skull. Sutures are the areas between the cranial bones that fuse during childhood. In diastatic fractures, these normal suture lines become widened. These fractures are more commonly seen in newborns and older infants.

Basilar skull fracture: basilar skull fractures are the most severe type of skull fracture and involve a break in the bone at the base of the skull. Patients with this type of fracture often display bruising around their eyes and behind their ears. Additionally, they may experience clear fluid drainage from their nose or ears due to damage to the brain's covering. Patients with basilar skull fractures typically require close observation in a hospital setting.

In the context of adult head injuries, an approach for the detection of skull fractures was developed, building upon the Faster R-CNN framework (Kuang et al., 2020). This method leveraged the morphological characteristics of the skull, introducing a skeleton-based region proposal technique to enhance the concentration of candidate detection boxes in critical regions while minimizing the occurrence of invalid boxes. This optimization allowed for the removal of the region proposal network within Faster R-CNN, reducing computational requirements. Additionally, a full-resolution feature network was crafted to extract features, rendering the model more attuned to detecting smaller objects. In comparison to prior methods for skull fracture detection, the authors observed a significant reduction in false positives while maintaining a high level of sensitivity.

Another study developed a deep learning system for automated identification of skull fractures from cranial CT scans (Shan et al., 2021). This study retrospectively analyzed CT scans of 4,782 male and female patients diagnosed with skull fractures between. Additional data of 7,856 healthy people were included in the analysis to reduce the probability of false detection. Skull fractures in all the scans were manually labeled by seven experienced neurologists. Two deep learning approaches were developed and tested for the identification of skull fractures. In the first approach, the fracture identification task was treated as an object detected problem, and a YOLOv3 network (Redmon et al., 2015) was trained to identify all the instances of skull fracture. In the second approach, the task was treated as a segmentation problem and a modified attention U-net (Ronneberger et al., 2015) was trained to segment all the voxels representing skull fracture. The developed models were tested using an external test set of 235 patients (93 with, and 142 without skull fracture). On the test set, the YOLOv3 achieved average fracture detection sensitivity and specificity of 81% and 86%, respectively. On the same dataset, the modified attention U-Net achieved a fracture detection sensitivity and specificity of 83% and 89%, respectively.

A recent investigation was conducted to assess the diagnostic capabilities of a deep learning algorithm [RetinaNet (Lin et al., 2020)] for the detection of skull fractures in plain radiographic images and to explore its clinical utility (Jeong et al., 2023). The study encompassed a dataset of 2,026 plain radiographic images of the skull, comprising 991 images with fractures and 1,035 images without fractures, gathered from 741 patients. The deep learning model employed for this purpose was the RetinaNet architecture. The authors observed significant disparities in the true/false and false-positive/false-negative ratios across different views, including the anterior-posterior and both lateral perspectives. Notably, false positives were often related to the detection of vascular grooves and suture lines, while false negatives exhibited suboptimal performance in detecting diastatic fractures, fractures crossing suture lines, and fractures around vascular grooves and the orbit.

In the context of pediatric head injuries, YOLOv3 (Redmon et al., 2015) was applied to the task of detecting skull fractures in children using plain radiographs (Choi et al., 2022). This retrospective, multi-center study incorporated a development dataset sourced from two hospitals (*n* = 149 and 264) and an external test set (*n* = 95) from a third medical facility. The datasets encompassed children who had experienced head trauma and had undergone both skull radiography and cranial CT scans. The study involved the participation of two radiology residents, a pediatric radiologist, and two emergency physicians in a two-phase observer study using an external test set, both with and without the assistance of AI. Results revealed an improvement in the area under the receiver operating characteristic curve (AUC) for radiology residents and emergency physicians when aided by the AI model, with improvements of 0.094 and 0.069, respectively, compared to readings performed without AI assistance. In contrast, the pediatric radiologist experienced a more modest improvement of 0.008. The findings indicate that the AI model can enhance the diagnostic performance of less experienced radiologists and emergency physicians when it comes to identifying pediatric skull fractures on plain radiographs.

## 6 Discussions and next steps

Applications of AI are everywhere. Although AI has been applied to perform imaging diagnostics and shown many show breakthroughs in medical imaging (Moawad et al., 2022), its limitations in fracture detection and classification have not been well investigated in the literature (Langerhuizen et al., 2019). The chronologically published works reviewed in this study, which are summarized in Table 2, illustrate that the integration of AI methods for the diagnosis and treatment planning of facial trauma is still in an early stage. Limitations and discussions for further investigation and development of AI-enabled imaging diagnostic systems are addressed in the subsequent sections.

**Table 2:** Chronological order of applications and developments of AI for facial fracture detection reviewed in this study.

**Year**	**References**	**Fracture types**	**Image modality**	**AI methods**
2018	Chilamkurthy et al. (2018)	Frontal-bone fractures	CT	ConvNets
2020	Kuang et al. (2020)	Skull	CT	Faster R-CNN
2021	Shan et al. (2021)	Skull	CT	YOLO and U-Net
2021	Ryu et al. (2021) (survey)	Cranial fractures	Photographs, CT	Pretrained CNNs, RNNs, GANs
2021	Amodeo et al. (2021)	Maxillofacial fractures	CT	Pretrained CNN
2022	Yang et al. (2022)	Nasal fractures	CT	Deep learning
2022	Seol et al. (2022)	Nasal fractures	CT	3D pretrained CNNs
2022	Moon et al. (2022)	Nasal fractures	CT	YOLOX
2022	Vinayahalingam et al. (2022)	Mandibular fractures	Panoramic radiographs	Faster R-CNN
2022	Wang et al. (2022)	Mandibular fractures	CT	Pretrained CNNs
2022	Son et al. (2022)	Mandibular fractures	Panoramic radiographs	U-Net, YOLO
2022	Choi et al. (2022)	Pediatric skull	CT	YOLOv3
2023	Warin et al. (2023)	Maxillofacial fractures	CT	Pretrained CNNs, Faster R-CNN, YOLO
2023	Wang et al. (2023)	Craninal and facial fractures	CT	YOLOv4, ResUNet++
2023	Jeong et al. (2023)	Skull	Plain radiographs	RetinaNet

### 6.1 Advantages and disadvantages of adopted AI models for facial and skull fracture detection

Applications of the AI models discussed in this review for the diagnosis of bone fractures in facial trauma present specific advantages and disadvantages, which are elaborated below (summary is given in Table 3).

**Table 3:** Summarized advantages and disadvantages of AI models for facial fracture detection.

**Model**	**Advantages**	**Disadvantages**
Pretrained CNNs	Feature extraction, reduced training time, generalization capability, transfer learning	Limited specificity, overfitting risks, need for fine-tuning, limited adaptability
RNNs	Sequential data analysis, memory retention, dynamic temporal modeling, contextual understanding	Vanishing gradients, training complexity, limited contextual memory, sequential processing limitations
GANs	Data augmentation, image enhancement and restoration, unsupervised learning and feature extraction, anomaly detection	Training instability, computational complexity, data bias and generalization challenges
Faster R-CNN	Accurate object localization, real-time detection, robust feature extraction, high performance on large datasets	Computational complexity, Limited generalization to unseen data, Training data requirements, Interpretability and explainability
YOLO versions	Real-time processing, high accuracy in object detection, efficient resource utilization, simplicity and ease of implementation	Limited sensitivity to small objects, challenges in handling overlapping objects, raining data requirements, generalization limitations
U-Net	Effective image segmentation, robust feature extraction, residual skip connections, adaptability to limited data	Computational complexity, limited generalization to unseen data, risk of overfitting, model interpretability
ResUNet++	Highly effective feature extraction, comprehensive image segmentation, residual connections for improved performance, adaptability to limited data	Computational complexity, risk of overfitting, model interpretability, limited generalization to unseen data
RetinaNet	Precise object detection, efficient handling of scale variation, high accuracy with dense object detection, effective training with imbalanced data	Computational complexity, limited generalization to unseen data, risk of overfitting with small datasets, model interpretability

#### 6.1.1 Pretrained CNNs

Advantages:

- Feature extraction: pre-trained CNNs can leverage their ability to extract intricate features from images, facilitating the identification of complex patterns and subtle details associated with different types of bone fractures in facial trauma.
- Reduced training time: by utilizing pre-trained CNNs, the need for extensive training on large datasets is minimized, thereby reducing the computational resources and time required for model development, enabling quicker deployment in clinical settings.
- Generalization capability: based on architectures that have been fine-tuned and optimized on large-scale datasets, pre-trained CNNs are adept at generalizing patterns from one domain to another, allowing for effective adaptation to various types of bone fractures in facial trauma, even in cases with limited training data.
- Transfer learning: pre-trained CNNs enable transfer learning, wherein knowledge acquired from one dataset or problem domain can be applied to a different but related problem, facilitating the development of accurate and reliable fracture diagnosis models even with limited labeled data.

Disadvantages:

- Limited specificity: pre-trained CNNs might not be specifically tailored for the diagnosis of bone fractures in facial trauma, leading to potential challenges in accurately capturing the intricate characteristics and variations associated with facial trauma injuries, thereby compromising diagnostic accuracy.
- Overfitting risks: there is a risk of overfitting when using pre-trained CNNs, especially when the pre-training dataset significantly differs from the target application domain, potentially leading to reduced generalizability and reliability in fracture diagnosis.
- Need for fine-tuning: fine-tuning pre-trained CNNs requires careful adjustments to ensure optimal performance for the specific task of diagnosing bone fractures in facial trauma, necessitating expertise in model adaptation and parameter optimization.
- Limited adaptability: pre-trained CNNs may struggle to adapt to rare or novel fracture patterns or cases that deviate significantly from the distribution of fractures seen in the pre-training dataset, potentially leading to misdiagnosis or overlooked critical cases.

#### 6.1.2 RNNs

Advantages:

- Sequential data analysis: RNNs are well-suited for analyzing sequential data, enabling the capture of temporal dependencies and patterns within medical imaging data, which can be beneficial in identifying complex fracture patterns and subtle variations in facial trauma injuries.
- Memory retention: RNNs possess the ability to retain information from previous inputs, making them effective in processing and analyzing long sequences of medical imaging data, thus aiding in the accurate diagnosis of bone fractures in facial trauma that might require a comprehensive analysis of multiple images or frames.
- Dynamic temporal modeling: RNNs can dynamically model temporal changes and variations in medical imaging data, allowing for a more nuanced understanding of the progression of fractures in facial trauma over time, thereby enhancing the diagnostic accuracy and predictive capabilities of the model.
- Contextual understanding: RNNs facilitate the development of models that can understand the contextual relationship between different components of medical imaging data, enabling a more comprehensive analysis of fracture patterns and their implications within the broader context of facial trauma diagnosis.

Disadvantages:

- Vanishing gradients: RNNs are susceptible to the issues of vanishing gradients, which can impede the training process and hinder the model's ability to effectively capture long-term dependencies and subtle patterns in the data, potentially leading to reduced diagnostic accuracy in complex fracture cases.
- Training complexity: training RNNs can be computationally intensive, especially when dealing with large datasets, complex fracture patterns, and high-resolution medical imaging data, requiring significant computational resources and time, which could limit the feasibility of real-time diagnosis in clinical settings.
- Limited contextual memory: some RNN variants suffer from limitations in capturing long-range dependencies and maintaining contextual memory over extended sequences, potentially leading to difficulties in comprehensively analyzing and diagnosing intricate fracture patterns and complex facial trauma cases.
- Sequential processing limitations: RNNs process data sequentially, which may limit their ability to simultaneously capture and analyze multiple aspects or features within medical imaging data, potentially leading to challenges in accurately identifying and diagnosing complex fracture patterns and associated trauma injuries.

#### 6.1.3 GANs

Advantages:

- Data augmentation: GANs can be utilized to generate synthetic medical imaging data, thereby augmenting limited training datasets and enhancing the robustness of fracture diagnosis models, especially in cases where obtaining large and diverse datasets of facial trauma injuries is challenging.
- Image enhancement and restoration: GANs can facilitate the enhancement and restoration of low-quality or noisy medical imaging data, improving the overall image quality and aiding in the accurate identification and diagnosis of intricate fracture patterns and subtle variations in facial trauma injuries.
- Unsupervised learning and feature extraction: GANs enable unsupervised learning, allowing for the extraction of meaningful features and representations from medical imaging data without the need for extensive labeled datasets, thereby facilitating the development of more comprehensive and accurate fracture diagnosis models.
- Anomaly detection: GANs can be employed for anomaly detection in medical imaging data, assisting in the identification of rare or complex fracture patterns and abnormalities that might be challenging to diagnose using conventional methods, thereby improving the overall diagnostic accuracy and precision.

Disadvantages:

- Training instability: GANs are prone to training instability, often leading to issues such as mode collapse, vanishing gradients, or oscillating convergence, which can hinder the model's ability to accurately capture and generate realistic fracture patterns in facial trauma, thereby affecting the diagnostic performance.
- Computational complexity: training GANs can be computationally intensive, demanding substantial computational resources and time, particularly when dealing with high-resolution medical imaging data and complex fracture patterns, potentially limiting the feasibility of real-time diagnosis and clinical deployment.
- Data bias and generalization challenges: GANs might encounter difficulties in generating diverse and representative synthetic medical imaging data, leading to potential biases and limitations in the generalization of the fracture diagnosis models to different patient populations or trauma scenarios, thereby affecting the overall diagnostic reliability.

#### 6.1.4 Faster R-CNN

Advantages:

- Accurate object localization: Faster R-CNN is effective in accurately localizing and identifying the precise locations of fractures within complex medical imaging data, facilitating the accurate diagnosis and assessment of various types of bone fractures in facial trauma with high precision.
- Real-time detection: Faster R-CNN can operate in real-time or near real-time, enabling swift and efficient fracture diagnosis, particularly in time-sensitive clinical scenarios and emergency situations, thereby facilitating prompt medical intervention and treatment planning for patients with facial trauma injuries.
- Robust feature extraction: Faster R-CNN incorporates robust feature extraction mechanisms, allowing for the comprehensive analysis of intricate fracture patterns and subtle variations in medical imaging data, enhancing the overall diagnostic accuracy and reliability of fracture detection in facial trauma cases.
- High performance on large datasets: Faster R-CNN demonstrates high performance on large-scale datasets, making it well-suited for processing and analyzing extensive collections of medical imaging data associated with various types of facial trauma injuries, thereby enhancing the overall diagnostic capabilities and generalizability of the model.

Disadvantages:

- Computational complexity: implementing Faster R-CNN can be computationally intensive, especially when dealing with high-resolution medical imaging data and complex fracture patterns, demanding significant computational resources and processing power, which could limit its practicality for deployment in resource-constrained clinical environments.
- Limited generalization to unseen data: Faster R-CNN might face challenges in generalizing to unseen or rare fracture patterns and variations in facial trauma, potentially leading to reduced diagnostic accuracy and reliability in cases that deviate significantly from the training dataset, thereby affecting the model's overall performance in clinical settings.
- Training data requirements: Faster R-CNN requires a substantial amount of labeled training data for accurate model training and validation, posing challenges in cases where obtaining large and diverse datasets of facial trauma injuries for training purposes is impractical or resource-prohibitive.
- Interpretability and explainability: Faster R-CNN's complex architecture might limit its interpretability and explainability, making it challenging for clinicians to understand the underlying decision-making process and the factors contributing to fracture diagnosis, potentially affecting the trust and acceptance of the model in clinical practice.

#### 6.1.5 YOLO models

Advantages:

- Real-time processing: YOLO models enable real-time processing and detection of fractures in facial trauma, facilitating rapid and timely diagnosis, particularly in critical medical scenarios where immediate intervention is crucial for patient care.
- High accuracy in object detection: YOLO models demonstrate high accuracy in object detection tasks, allowing for precise identification and localization of fractures within complex medical imaging data, thereby enhancing the overall diagnostic precision and reliability.
- Efficient resource utilization: YOLO models optimize resource utilization, requiring fewer computational resources compared to other complex deep learning architectures, which can contribute to faster inference times and improved efficiency in fracture diagnosis workflows.
- Simplicity and ease of implementation: YOLO models are relatively straightforward to implement and deploy, making them accessible to a broader range of healthcare practitioners and institutions, thereby facilitating their integration into clinical settings for efficient and accurate fracture diagnosis in facial trauma cases.

Disadvantages:

- Limited sensitivity to small objects: YOLO models may demonstrate reduced sensitivity to small or subtle fracture patterns in facial trauma, potentially leading to missed detections or inaccuracies in the diagnosis of minor fractures, which could impact the overall diagnostic reliability of the model.
- Challenges in handling overlapping objects: YOLO models might encounter challenges in accurately distinguishing and delineating overlapping fractures or complex fracture patterns within medical imaging data, potentially leading to misinterpretations or errors in the diagnosis of overlapping facial trauma injuries.
- Training data requirements: YOLO models require substantial amounts of labeled training data to achieve optimal performance, posing challenges in cases where obtaining diverse and comprehensive datasets of facial trauma injuries for model training and validation is impractical or resource-intensive.
- Generalization limitations: YOLO models might face difficulties in generalizing to unseen or rare fracture patterns and variations in facial trauma, potentially leading to reduced diagnostic accuracy and reliability in cases that deviate significantly from the training dataset, thereby affecting the model's overall performance in clinical settings.

#### 6.1.6 U-Net

Advantages:

- Effective image segmentation: U-Net models excel in image segmentation tasks, enabling precise delineation and segmentation of fractured bone structures within complex medical imaging data, thereby enhancing the overall accuracy and reliability of fracture diagnosis in facial trauma cases.
- Robust feature extraction: U-Net models facilitate robust feature extraction, allowing for a comprehensive analysis of intricate fracture patterns and subtle variations in facial trauma injuries, enhancing the diagnostic capabilities of the model and aiding in the accurate identification of complex fracture patterns.
- Residual skip connections: U-Net models utilize residual skip connections, which enable the seamless integration of low-level and high-level features, enhancing the model's ability to capture both local and global information within medical imaging data, thereby improving the overall diagnostic precision and performance.
- Adaptability to limited data: U-Net models can effectively adapt to limited training data, making them well-suited for scenarios where obtaining extensive and diverse datasets of facial trauma injuries for model training and validation is challenging or resource-prohibitive.

Disadvantages:

- Computational complexity: implementing U-Net models can be computationally intensive, especially when dealing with high-resolution medical imaging data and complex fracture patterns, demanding substantial computational resources and processing power, which could limit the feasibility of real-time diagnosis and clinical deployment.
- Limited generalization to unseen data: U-Net models might encounter challenges in generalizing to unseen or rare fracture patterns and variations in facial trauma, potentially leading to reduced diagnostic accuracy and reliability in cases that deviate significantly from the training dataset, thereby affecting the model's overall performance in clinical settings.
- Risk of overfitting: U-Net models are susceptible to overfitting, particularly when the model is trained on small or imbalanced datasets, leading to reduced generalizability and potentially compromising the diagnostic accuracy and reliability of the model in real-world clinical applications.
- Model interpretability: U-Net models' complex architecture may pose challenges in model interpretability and explainability, making it difficult for clinicians to understand the underlying decision-making process and factors contributing to fracture diagnosis, potentially affecting the trust and acceptance of the model in clinical practice.

#### 6.1.7 ResUNet++

Advantages:

- Highly effective feature extraction: ResUNet++ models leverage residual connections and U-Net architecture, enabling highly effective feature extraction and representation learning, which aids in the precise identification and segmentation of intricate fracture patterns and bone structures within medical imaging data.
- Comprehensive image segmentation: ResUNet++ models excel in comprehensive image segmentation tasks, facilitating the accurate delineation and segmentation of complex fracture patterns and subtle variations in facial trauma injuries, thereby enhancing the overall diagnostic accuracy and reliability of fracture diagnosis.
- Residual connections for improved performance: ResUNet++ models utilize residual connections to integrate skip connections and shortcut connections, enhancing the model's ability to capture both local and global features within medical imaging data, leading to improved performance and robustness in fracture diagnosis.
- Adaptability to limited data: ResUNet++ models can adapt to limited training data, making them suitable for scenarios where acquiring extensive and diverse datasets of facial trauma injuries for model training and validation is challenging or resource-prohibitive.

Disadvantages:

- Computational complexity: implementing ResUNet++ models can be computationally intensive, particularly when dealing with high-resolution medical imaging data and complex fracture patterns, demanding significant computational resources and processing power, which could limit the feasibility of real-time diagnosis and clinical deployment.
- Risk of overfitting: ResUNet++ models are susceptible to overfitting, especially when trained on small or imbalanced datasets, which may reduce the generalizability and compromise the diagnostic accuracy and reliability of the model in real-world clinical applications.
- Model interpretability: ResUNet++ models' complex architecture may pose challenges in model interpretability and explainability, making it difficult for clinicians to understand the underlying decision-making process and factors contributing to fracture diagnosis, potentially affecting the trust and acceptance of the model in clinical practice.
- Limited generalization to unseen data: ResUNet++ models may face challenges in generalizing to unseen or rare fracture patterns and variations in facial trauma, potentially leading to reduced diagnostic accuracy and reliability in cases that significantly deviate from the training dataset, impacting the model's overall performance in clinical settings.

#### 6.1.8 RetinaNet

Advantages:

- Precise object detection: RetinaNet models excel in precise object detection tasks, enabling accurate identification and localization of fractures within complex medical imaging data, thereby enhancing the overall diagnostic precision and reliability in the context of facial trauma diagnosis.
- Efficient handling of scale variation: RetinaNet models efficiently handle scale variation in medical imaging data, allowing for the effective detection of fractures of varying sizes and complexities within facial trauma injuries, thereby improving the model's adaptability to diverse fracture patterns.
- High accuracy with dense object detection: RetinaNet models demonstrate high accuracy in dense object detection tasks, facilitating the comprehensive analysis of intricate fracture patterns and subtle variations in facial trauma injuries, leading to improved diagnostic capabilities and robust fracture detection.
- Effective training with imbalanced data: RetinaNet models are effective in training with imbalanced datasets, making them suitable for scenarios where obtaining balanced datasets of facial trauma injuries for model training and validation is challenging, thereby enhancing the model's generalizability and performance.

Disadvantages:

- Computational complexity: implementing RetinaNet models can be computationally intensive, particularly when dealing with high-resolution medical imaging data and complex fracture patterns, demanding significant computational resources and processing power, which could limit the feasibility of real-time diagnosis and clinical deployment.
- Limited generalization to unseen data: RetinaNet models might face challenges in generalizing to unseen or rare fracture patterns and variations in facial trauma, potentially leading to reduced diagnostic accuracy and reliability in cases that significantly deviate from the training dataset, impacting the model's overall performance in clinical settings.
  Risk of overfitting with small datasets: RetinaNet models are susceptible to overfitting, especially when trained on small or imbalanced datasets, which may reduce the model's generalizability and compromise the diagnostic accuracy and reliability of fracture detection in real-world clinical applications.
- Model interpretability: RetinaNet models' complex architecture may pose challenges in model interpretability and explainability, making it difficult for clinicians to understand the underlying decision-making process and factors contributing to fracture diagnosis, potentially affecting the trust and acceptance of the model in clinical practice.

### 6.2 Limitations of current approaches

Despite the advancements in AI for the diagnosis of bone fractures in facial trauma, several limitations persist within the current approaches. Addressing the following limitations requires continued research, collaboration between AI experts and clinicians, and the development of robust frameworks for data collection, model training, validation, and legal implementation, ultimately ensuring the safe and effective integration of AI in the diagnosis of bone fractures in facial trauma.

Data limitations and diversity: many AI models are trained on limited and homogeneous datasets, potentially leading to biased results and reduced generalizability. The lack of diverse datasets, comprising various ethnicities, age groups, and trauma mechanisms, hinders the ability of AI systems to accurately diagnose fractures in a broader patient population.

Complex fracture patterns: AI systems may face challenges in accurately identifying complex fracture patterns, especially those that involve multiple bone structures or intricate facial trauma. Current AI models might not fully capture the nuanced variations in fracture presentations, leading to potential inaccuracies or missed diagnoses in complex cases.

Interpretability and explainability: some AI models used in fracture diagnosis lack transparency in their decision-making process (see Table 3), making it challenging for clinicians to understand how the AI arrives at a specific diagnosis. The lack of interpretability and explainability can undermine trust in AI-generated results and hinder the integration of AI technology into clinical workflows.

Legal concerns: the use of AI in fracture diagnosis raises legal concerns regarding liability in case of misdiagnosis. Ensuring compliance with regulatory standards remains a critical challenge for the widespread implementation of AI in facial trauma diagnosis.

Dependency on imaging quality: AI models heavily rely on the quality of imaging data for accurate fracture detection. Poor image quality, artifacts, or technical limitations in imaging techniques can significantly impact the performance of AI algorithms, leading to potential diagnostic errors or false interpretations.

Validation and clinical integration: adequate validation and integration of AI systems into clinical practice remain crucial challenges. Limited clinical validation studies and the absence of standardized protocols for AI implementation in fracture diagnosis hinder the widespread adoption of AI technology by healthcare institutions and professionals.

### 6.3 General limitations

In general, standards for training and testing an AI-enabled system for recognizing fractures in images are still needed for integrating into routine emergency workflows; applications of AI to more complex fracture diagnostics with wider age subgroups; addressing legal regulations; and identification of feasible implementation in clinical settings (Langerhuizen et al., 2019; Parpaleix et al., 2023).

Errors occurred in diagnosis of fractures in emergency departments result in wrong, neglected, or delayed findings (Graber, 2005). It has been suggested that there are four main legal causes concerning radiologists, which are errors in (1) observation and (2) interpretation, failures of (3) suggesting the next appropriate procedure and (4) a timely and clinically appropriate communication (Pinto and Brunese, 2010; Pinto et al., 2018). Spectrum of fractures that are potentially missed on the reading of images, including various modalities, by radiologists are not comprehensively addressed in the applications of AI for image analysis of bone fractures. Recent comparisons between radiologists, AI-assisted radiologists, and independent AI-based diagnostic systems for detecting bone fractures in trauma emergency have shown the potential of AI for leveraging human performance and productivity (Canoni-Meynet et al., 2022). Such AI-assisted diagnostics can be very useful for emergent fracture diagnosis in the late and early hours during which misdiagnosed fractures are at peak (Hallas and Ellingsen, 2006). However, the study was limited to a simple case of binary detection and without an inclusion of fracture variety. Because fracture variability is of high clinical relevance, there is a need to investigate the value of AI in identification of dislocated fractures vs. non-dislocated fractures.

Three-dimensional imaging techniques have been increasingly used in dentistry (Hung e al., 2020). Dental 3D-imaging are useful for evaluating severe facial injury as it can provides a clear view of major fracture lines and displaced fragments. Particularly for maxillofacial trauma, the capability of 3D-imaging enables better preoperative analysis and surgical planning than conventional radiography (Kaur and Chopra, 2010). Multi-detector CT (MDCT) has been reported in the literature as the gold-standard imaging modality for facial bones (Hooper et al., 2019). However, MDCT applies a high radiation dose to the patient. This gives rise to the importance for developing dose reduction and dose optimisation methods in MDCT (Hooper et al., 2019). At the same time, this modality also gives rise to the development of AI-based tools for research into facial trauma, where dental cone beam CT, intraoral, and facial scans can be reconstructed into 3D visuals to be analyzed by machine learning for improving diagnosis, enhancing treatment planning, and providing accurate prediction of treatment outcome (Hung e al., 2020).

### 6.4 Data availability

In comparison, AI applications to facial trauma are far less reported in literature than many other fields of medicine such as cancerous, neurodegenerative, and cardiovascular diseases. A main reason is the lack of access to public data to allow AI researchers further explore applications of advanced models for image analysis and detection of fractures in facial trauma. Up to date, almost publications, including open-access articles, have indicated that data used the studies are not publicly available due to data protection, or are available from corresponding authors on reasonable requests. Thus, the need for getting access to documented image databases of facial trauma in public depositories is imperative to advance and keep pace of the research into imaging diagnostics of facial trauma with other areas of medical research. Here, it is pointed out that The National Maxillofacial Surgery Unit of Ireland has recently announced its effort in providing a prospective maxillofacial trauma database to facilitate several important aspects such as strategic planning, resource allocations, clinical compliance, auditing, and research (Henry et al., 2022).

### 6.5 Data imbalance

Addressing imbalanced classes in image-based diagnosis of fractures in patients with facial trauma is crucial, as the inherent bias in AI-based solutions can lead to suboptimal performance and misdiagnoses. Imbalanced classes occur when one class (e.g., non-fractured) significantly outweighs the other (e.g., fractured) in the dataset, causing machine learning models to favor the majority class. Here are some key issues related to imbalanced classes in this context and techniques to mitigate them:

Data collection bias: the data used to train AI models may be biased toward non-fractured cases because they are more prevalent. This bias can lead to models that are less sensitive to fractures, as they might not have enough exposure to the minority class. Efforts should be made to collect a diverse and representative dataset that includes a sufficient number of fractured cases. This may involve collaboration with multiple healthcare institutions to ensure a more balanced dataset.

Model performance: imbalanced classes can lead to models with high accuracy but poor sensitivity, meaning they may perform well on non-fractured cases but miss fractures. Several techniques can help address this issue, including resampling methods (oversampling the minority class or undersampling the majority class), using different evaluation metrics like F1-score or AUC, and adjusting class weights during model training to give more importance to the minority class.

Data augmentation: in cases where collecting more data is challenging, data augmentation techniques can help balance the dataset. For image-based fracture diagnosis, this might involve generating synthetic images of fractures by applying transformations to existing fractured images. Augmentation techniques such as rotation, translation, scaling, and introducing random noise can be applied to the minority class to increase its size and diversity, making the model more robust.

Transfer learning: leveraging pre-trained models trained on large, diverse datasets can be beneficial. However, these models may also be biased toward the majority class. Fine-tuning the pre-trained models on the imbalanced dataset while using techniques like class-weighting can help adapt them to the specific task of fracture diagnosis.

Ensemble methods: combining multiple models or classifiers, especially those designed to handle imbalanced data, can improve performance. Ensemble techniques like bagging, boosting, or stacking can help create a more robust and balanced diagnostic system by combining the outputs of multiple classifiers.

Active learning: in an iterative process, AI models can actively query additional data points that are uncertain or challenging, potentially focusing on underrepresented fracture cases to improve overall performance. Active learning strategies can help in selecting the most informative samples for human review and annotation, gradually improving the model's performance in recognizing fractures.

In general, addressing the issue of imbalanced classes in image-based fracture diagnosis is essential to ensure that AI-based solutions are equitable and clinically effective. It requires a combination of thoughtful data collection, algorithmic techniques, and model evaluation strategies to mitigate bias and improve diagnostic accuracy for both fractured and non-fractured cases. Moreover, continuous monitoring and updates to the model as more data becomes available can help maintain its diagnostic performance over time.

### 6.6 Pediatric facial trauma

It has been reported that craniofacial trauma is common in children, where most incidences involved soft tissue and dentoalveolar damages (Braun et al., 2017). Although being relatively rare in comparison with the adult population, pediatric facial trauma can cause significant morbidity and disability. The diagnosis and treatment of pediatric facial trauma can be different from adult facial trauma because the former procedures depend on stages of development and future growth, which yield different patterns of injury (Braun et al., 2017).

Studies reviewed in the foregoing sections on the applications of AI were mostly about fracture detection in adults. Little effort has been made on developing AI-based methods for detecting and classifying images of pediatric bone fractures in general and facial fractures in particular, despite the long-term clinical consequences of failure to diagnose fractures in children. Shelmerdine et al. (2022) reviewed the use of AI for radiological pediatric fracture assessment. The authors pointed out differences of patterns between pediatric and adult bone-fracture appearances on imaging, which led to the missing of up to 11% of misdiagnosis of acute pediatric fractures performed by emergency physicians compared to specialist pediatric radiologists. Some highlights provided by the authors are that AI-based tools for imaging diagnostics of facial trauma in children were developed to mostly address assessments on plain radiography. The AI methods were constrained with strict inclusion and exclusion criteria that would make it difficult to apply to pediatric cases. Opportunities for important future research suggested by the authors include the development of AI-enabled fracture assessment for very young children, who are <2 years of age, and children with genetic bone disorders.

### 6.7 Features for image classification

Although CNNs are capable of extracting useful features from images through a series of convolution, nonlinearity, and pooling; exploration and inclusion of novel features extracted from raw image data to be used as input into a deep-learning model can enhance the classification power of the AI method. Alternatively, image features extracted with deep learning can be further mapped into a different type of features that are expected to increase the similarity of images that belong to the same class and dissimilarity between those that belong to different classes. For examples, the transformation of raw images into images of pixel recurrence could enhance the deep learning of complex spatial information in immunohistochemical (IHC) images for the prognosis of patients with rectal cancer (Pham et al., 2023a); and the use of wavelet-image scattering for the second feature extraction of IHC features obtained from a pretrained CNN could reveal the prognostic power of a protein marker in rectal cancer (Pham and Sun, 2023).

The fusion of image features extracted from multiple pretrained CNNs would be useful for improving the detection of complex fractures in facial trauma. Pretrained CNNs self-extract features of images at different layer activations along its network depth. Activation-based features obtained multiple pretrained CNNs can be readily adopted for machine learning such as a support vector machine (SVM) for object classification. Some good reasons for using image features from pretrained CNNs to train an SVM are that the feature extraction process performed by a pretrained CNN is computationally efficient and CNN-based features have been known to be more effective for SVM-based classification than the direct use of a pretrained CNN-based classifier (Fan et al., 2021; Pham et al., 2023b). This practice is particularly useful if the size of a dataset of facial trauma is too small to enable an effective adoption of the transfer learning with a pretrained CNN (Fan et al., 2021; Pham et al., 2023b). It is because the fine-tuning of pretrained deeper layers does not likely improve the differentiation of class features when there is not sufficient information to learn from a small dataset.

### 6.8 Evidence-based decision

Scarfe (2005) pointed out that there has been no general agreement of diagnostic imaging selection in maxillofacial trauma. Complexity and economic tradeoffs between technologies, healthcare reimbursement, and legal issues interplay contribute to local selection approaches. The author also reported that while 3D images are the preferred modality, it may lead to different surgical approach and determination of clinical diagnostic values. These discrepancies are the results of different interpretations on the radiologic information and the nature of the trauma provided by different image readers. Therefore, the combination of evidence-based clinical decision making and radiographic selection criteria for addressing appropriate radiographic modality in facial trauma is necessary. AI-based clinical decision support systems can offer potential benefits to this development as its similar applications have been reported in literature (Giordano et al., 2021; Amann et al., 2022; Ramgopal et al., 2023 ).

Generally, evidence-based medicine (EBM) encompasses the evidence from clinical studies, medical expertise, and the patient's values and preferences for clinical decision making (Sackett et al., 1996). The principles of EBM have led to a systematic approach to clinical problem solving (Akobeng, 2005). However, it is reported that EBM has encountered several difficulties in implementation, known as the “60-30-10 Challenge” (Braithwaite et al., 2020). Nowak (2021) has recently suggested potential solutions to this challenge with the use of AI, including its ability to (1) detect research gaps and improve the allocation of medical research funds, (2) accelerate the process of evidence synthesis, and (3) automate the living systematic reviews. Such suggestions are applicable to evidence-based decision making in facial trauma imaging.

### 6.9 AI overreliance

It was thought that a human-AI interaction system could perform better than either a human-based or an AI-based system. However, several investigations have reported that this is not always true. It is observed that humans tend to accept outputs or decisions made by AI or machine-learning algorithms. This problem is known as AI or machine-learning overreliance. AI overreliance should be avoided in medical diagnoses such as the case of facial trauma. Because the way AI processes information is complex and considered as a black box, explainable AI (Gunning et al., 2019) aims to interpret predictions made by machine-learning models and help reduce overreliance on AI.

A recent experimental study (Vasconcelos et al., 2023) has illustrated that humans can overcome AI overreliance in making decisions when the accompanying AI explanations become simpler than the process of making decisions or they are motivated with monetary rewards for making correct decisions or predictions. But when facing challenging tasks, the authors found that explainable AI tools failed to help human users to reduce overreliance on AI because the complexity of the provided explanations was of the same level as the tasks themselves. Thus, while explainable AI is to be improved by the AI research community to elucidate AI mechanisms for making predictions, resulting in error reduction with a human-AI interaction, it is important for emergency radiologists and surgeons to have the knowledge of this current issue with AI technology.

In the context of AI overreliance, Rubin (2019) pointed out the role of radiologists in imaging AI. The author suggested precautionary measures for engagement with imaging AI-based decision tools: radiologists need to (1) consider the appropriate use of AI in specific clinical use cases, (2) be able to evaluate the adopted AI software, and (3) be informed of possible errors as a result of overreliance on AI tools.

The scientific community has been actively addressing the challenges of AI overreliance in medical applications to ensure that the integration of AI technology in healthcare remains safe, effective, and ethical. Some efforts include:

Developing robust validation protocols: scientists are working on establishing standardized and rigorous validation protocols to assess the performance and reliability of AI algorithms in medical applications (Tsopra et al., 2021). These protocols aim to ensure that AI systems are thoroughly evaluated before their deployment in clinical settings, minimizing the risk of relying on flawed or biased models.

Enhancing interpretability and explainability: researchers are focusing on improving the interpretability and explainability of AI models in healthcare (Markus et al., 2021). By developing techniques that provide insights into the decision-making process of AI algorithms, scientists aim to foster trust among healthcare professionals and patients, allowing them to understand how AI-generated recommendations are generated and making it easier to identify potential errors or biases.

Promoting ethical AI frameworks: the scientific community is actively advocating for the integration of ethical frameworks in the development and implementation of AI technologies in medicine (Gerke et al., 2020). Initiatives such as the development of guidelines for responsible AI use and the establishment of ethical review boards aim to ensure that AI applications prioritize patient safety, privacy, and data security while promoting transparency and accountability in the decision-making process.

Fostering collaboration between clinicians and AI experts: scientists are encouraging collaboration between clinicians and AI experts to ensure that AI solutions are designed to complement clinical workflows effectively (Wilson and Daugherty, 2018). By fostering interdisciplinary partnerships, the scientific community aims to develop AI technologies that are tailored to address specific clinical needs, improve diagnostic accuracy, and enhance treatment efficacy while taking into account the nuances and complexities of patient care.

Addressing bias and diversity: researchers are actively addressing issues related to bias and diversity in AI datasets used for medical applications (Norori et al., 2021). Efforts to improve the diversity of training data and mitigate biases within AI models aim to ensure that AI algorithms provide equitable and inclusive healthcare solutions that cater to diverse patient populations, thereby reducing disparities in healthcare delivery.

Overall, these concerted efforts underscore the scientific community's commitment to ensuring that AI remains a supportive tool in healthcare, assisting clinicians in making more informed decisions while upholding the highest standards of safety, reliability, and ethical practice.

### 6.10 Forensic medicine

AI has the potential to revolutionize the field of forensic medicine and pathology (Piraianu et al., 2023), especially in the diagnosis of fractures in facial trauma, aiding in the investigation and determination of the cause of violent deaths. Some potential applications of AI in this context are as follows.

Automated fracture detection and analysis: AI algorithms can be trained to automatically detect and analyze fractures in facial trauma from radiological images such as X-rays, CT scans, and MRIs. By leveraging deep learning and computer vision techniques, AI can accurately identify and classify different types of fractures, including their patterns and locations, which can provide valuable insights for forensic pathologists and investigators.

Pattern recognition for injury assessment: AI can be utilized to recognize specific patterns of facial fractures that may indicate particular types of violence or trauma. By analyzing a large dataset of facial trauma cases, AI models can learn to distinguish between fractures caused by different forces, such as blunt force trauma, gunshot wounds, or other types of assaults, aiding forensic experts in understanding the nature and potential source of the injury.

Comparative analysis and database integration: AI can facilitate the comparison of fracture patterns in facial trauma with a comprehensive database of known injury types, causes, and circumstances. By cross-referencing and analyzing a wide range of forensic data, including historical case records and relevant medical literature, AI systems can assist in identifying similarities or connections between current and past cases, potentially helping to establish patterns or identify potential suspects or weapons involved in the violence.

Simulation and reconstruction of trauma events: AI-powered simulations can reconstruct the sequence of events leading to the facial trauma, providing a visual representation of the force and impact that caused the fractures. By integrating data from various sources, including medical imaging, forensic evidence, and biomechanical principles, AI can help create virtual scenarios that depict how specific injuries might have occurred, assisting forensic experts in building more accurate and detailed narratives surrounding the circumstances of the violent death.

Data-driven forensic decision support systems: AI can contribute to the development of decision support systems that assist forensic pathologists and investigators in interpreting complex forensic data related to facial trauma. These systems can provide evidence-based insights and recommendations based on the analysis of extensive datasets, enabling more informed decision-making processes and potentially expediting the forensic investigation of violent deaths.

## 7 Conclusion

Recent developments of AI and its subset of deep learning for automated fracture detection in facial trauma imaging have been reviewed. These AI tools can be helpful for recognizing complex or subtle fractures. AI-enabled technology can help significantly reducing radiologic reading time in complex cases to allow specialists perform more essential tasks in an emergency department (Guermazi et al., 2022). Although further studies are required for model improvement and validation with randomized clinical trials with diverse samples of patients, AI-based predictive software and systems are promising for translation into clinical practice (Oppenheimer et al., 2023).

The concept of personalized and precision medicine (Hodson, 2016) departs from the conventional one-size-fits-all approach in medicine that is only effective for the average population of patients. Precision medicine (Hodson, 2016) is a modern medical innovation that considers individual differences in molecular characteristics, environments, and lifestyles of individual patients. AI has been playing a critical role for making precision medicine and health care practically achievable (Uddin et al., 2019; Johnson et al., 2021). Likewise, precision dentistry or dental medicine (Schwendicke and Krois, 2022; Kaur et al., 2023) is a novel data-driven approach to oral and dental health that tries to gain insights into the individual patient's genetics to deliver precise or targeted treatment. AI is believed to be central to the reality of precision dental medicine. AI for maxillofacial imaging is an obvious case of precision dental medicine, where big data of digital dental images can be readily analyzed by AI algorithms for precise object detection and segmentation to localize pathology, and accurate diagnosis of abnormal conditions (Hung et al., 2023).

This review is expected to be of clinical impact because AI and its machine-learning algorithms can offer effective and efficient solutions to challenging medical imaging problems in facial trauma, which are currently not well explored, to improve existing clinical reporting systems and lead to better patient outcomes.

## Author contributions

TP: Conceptualization, Investigation, Writing—original draft, Writing—review & editing. SH: Investigation, Writing—review & editing. PC: Investigation, Writing—review & editing.

## References

[B1] Ajmera P. Kharat A. Botchu R. Gupta H. Kulkarni V. (2021). Real-world analysis of artificial intelligence in musculoskeletal trauma. J. Clin. Orthop. Trauma 22, 101573. 10.1016/j.jcot.2021.101573 34527511 PMC8427222

[B2] Akobeng A. K. (2005). Principles of evidence based medicine. Arch. Dis. Child. 290, 837–840. 10.1136/adc.2005.071761 PMC1720507 16040884

[B3] Amann J. Vetter D. Blomberg S. N. Christensen H. C. Coffee M. Gerke S.. (2022). To explain or not to explain?–artificial intelligence explainability in clinical decision support systems. PLOS Digit. Health 1, e0000016. 10.1371/journal.pdig.0000016 36812545 PMC9931364

[B4] Amodeo M. Abbate V. Arpaia P. Cuocolo R. Dell'Aversana Orabona G. Murero M.. (2021). Transfer learning for an automated detection system of fractures in patients with maxillofacial trauma. Appl. Sci. 11, 6293. 10.3390/app11146293

[B5] Atighechi S. Karimi G. (2009). Serial nasal bone reduction: a new approach to the management of nasal bone fracture. J. Craniofac. Surg. 20, 49–52. 10.1097/SCS.0b013e318190def5 19164988

[B6] Bluthgen C. Becker A. S. Vittoria de Martini I. Meier A. Martini K. Frauenfelder T. (2020). Detection and localization of distal radius fractures: deep learning system versus radiologists. Eur. J. Radiol. 126, 108925. 10.1016/j.ejrad.2020.108925 32193036

[B7] Bocchialini G. Castellani A. (2019). Facial trauma: a retrospective study of 1262 patients. Ann. Maxillofac. Surg. 9, 135–139. 10.4103/ams.ams_51_19 31293942 PMC6585199

[B8] Bousson V. Attané G. Benoist N. Perronne L. Diallo A. Hadid-Beurrier L.. (2023). Artificial intelligence for detecting acute fractures in patients admitted to an emergency department: real-life performance of three commercial algorithms. Acad. Radiol. 30, 2118–2139. 10.1016/j.acra.2023.06.016 37468377

[B9] Braithwaite J. Glasziou P. Westbrook J. (2020). The three numbers you need to know about healthcare: the 60-30-10 challenge. BMC Med. 18, 102. 10.1186/s12916-020-01563-4 32362273 PMC7197142

[B10] Braun T. L. Xue A. S. Maricevich R. S. (2017). Differences in the management of pediatric facial trauma. Semin. Plast. Surg. 31, 118–122. 10.1055/s-0037-1601380 28496392 PMC5423796

[B11] Canoni-Meynet L. Verdot P. Danner A. Calame P. Aubry S. (2022). Added value of an artificial intelligence solution for fracture detection in the radiologist's daily trauma emergencies workflow. Diagn. Interv. Imaging 103, 594–600. 10.1016/j.diii.2022.06.004 35780054

[B12] Cha Y. Kim J.-T. Park C.-H. Kim J.-W. Lee S. Y. Yoo J.-I.. (2022). Artificial intelligence and machine learning on diagnosis and classification of hip fracture: systematic review. J. Orthop. Surg. Res. 17, 520. 10.1186/s13018-022-03408-7 36456982 PMC9714164

[B13] Cheng C.-T. Ho T.-Y. Lee T.-Y. Chang C.-C. Chou C.-C. Chen C.-C.. (2019). Application of a deep learning algorithm for detection and visualization of hip fractures on plain pelvic radiographs. Eur. Radiol. 29, 5469–5477. 10.1007/s00330-019-06167-y 30937588 PMC6717182

[B14] Cheng C.-T. Wang Y. Chen H.-W. Hsiao P.-M. Yeh C.-N. Hsieh C.-H.. (2021). A scalable physician-level deep learning algorithm detects universal trauma on pelvic radiographs. Nat. Commun. 12, 1066. 10.1038/s41467-021-21311-3 33594071 PMC7887334

[B15] Chilamkurthy S. Ghosh R. Tanamala S. Biviji M. Campeau N. G. Venugopal V. K.. (2018). Deep learning algorithms for detection of critical findings in head CT scans: a retrospective study. Lancet 392, 2388–2396. 10.1016/S0140-6736(18)31645-3 30318264

[B16] Choi J. W. Cho Y. J. Ha J. Y. Lee Y. Y. Koh S. Y. Seo J. Y.. (2022). Deep learning-assisted diagnosis of pediatric skull fractures on plain radiographs. Korean J. Radiol. 23, 343–354. 10.3348/kjr.2021.0449 35029078 PMC8876653

[B17] Chung K. J. Kim Y. H. Kim T. G. Lee J. H. Lim J. H. (2013). Treatment of complex facial fractures: clinical experience of different timing and order. J. Craniofac. Surg. 24, 216–220. 10.1097/SCS.0b013e318267b6f7 23348288

[B18] Dankelman L. H. M. Schilstra S. I. Jpma F. F. A. Doornberg J. N. Colaris J. W. Verhofstad M. H. J. (2022). Artificial intelligence fracture recognition on computed tomography: review of literature and recommendations. Eur. J. Trauma Emerg. Surg. 49, 681–691. 10.1007/s00068-022-02128-1 36284017 PMC10175338

[B19] Donat T. L. Endress C. Mathog R. H. (1998). Facial fracture classification according to skeletal support mechanisms. Arch. Otolaryngol. Head Neck Surg. 124, 1306–1314. 10.1001/archotol.124.12.1306 9865751

[B20] Dreizin D. Nam A. J. Diaconu S. C. Bernstein M. P. Bodanapally U. K. Munera F.. (2018). Multidetector CT of midfacial fractures: classification systems, principles of reduction, and common complications. Radiographics 38, 248–274. 10.1148/rg.2018170074 29320322

[B21] Duron L. Ducarouge A. Gillibert A. Lainé J. Allouche C. Cherel N.. (2021). Assessment of an AI aid in detection of adult appendicular skeletal fractures by emergency physicians and radiologists: a multicenter cross-sectional diagnostic study. Radiology 300, 120–129. 10.1148/radiol.2021203886 33944629

[B22] Esteva A. Robicquet A. Ramsundar B. Kuleshov V. DePristo M.. (2019). A guide to deep learning in healthcare. Nat. Med. 25, 24–29. 10.1038/s41591-018-0316-z 30617335

[B23] Expert Panel on Neurological Imaging Parsons, M. S. Policeni B. Juliano A. F. Agarwal M. Benjamin E. R.. (2022). ACR appropriateness criteria imaging of facial trauma following primary survey. J. Am. Coll. Radiol. 19, S67–S86. 10.1016/j.jacr.2022.02.013 35550806

[B24] Facial Fractures (2020). Cleveland Clinic, Cleveland, Ohio. Available online at: https://my.clevelandclinic.org/health/diseases/16025-facial-fractures (accessed March 30, 2023).

[B25] Fan J. Lee J. Lee Y. (2021). A transfer learning architecture based on a support vector machine for histopathology image classification. Appl. Sci. 11, 6380. 10.3390/app11146380

[B26] Gao Y. Soh N. Y. T. Liu N. Lim G. Ting D. Cheng L. T.-E.. (2023). Application of a deep learning algorithm in the detection of hip fractures. iScience 26, 107350. 10.1016/j.isci.2023.107350 37554447 PMC10404720

[B27] Gerke S. Minssen T. Cohen G. (2020). Ethical and legal challenges of artificial intelligence-driven healthcare. Artif. Intell. Healthcare 2020, 295–336. 10.1016/B978-0-12-818438-7.00012-5

[B28] Giordano C. Brennan M. Mohamed B. Rashidi P. Modave F. Tighe P.. (2021). Accessing artificial intelligence for clinical decision-making. Front Digit Health 3, 645232. 10.3389/fdgth.2021.645232 34713115 PMC8521931

[B29] Graber M. (2005). Diagnostic errors in medicine: a case of neglect. Jt. Comm. J. Qual. Patient Saf. 31, 106–113. 10.1016/S1553-7250(05)31015-4 15791770

[B30] Guermazi A. Tannoury C. Kompel A. J. Murakami A. M. Ducarouge A. Gillibert A.. (2022). Improving radiographic fracture recognition performance and efficiency using artificial intelligence. Radiology 302, 627–636. 10.1148/radiol.210937 34931859

[B31] Gunning D. Stefik M. Choi J. Miller T. Stumpf S. Yang G. Z.. (2019). XAI-explainable artificial intelligence. Sci. Robot. 4, eaay7120. 10.1126/scirobotics.aay7120 33137719

[B32] Hallas P. Ellingsen T. (2006). Errors in fracture diagnoses in the emergency department–characteristics of patients and diurnal variation. BMC Emerg. Med. 6, 4. 10.1186/1471-227X-6-4 16483365 PMC1386703

[B33] Head Injury (2023). Johns Hopkins Medicine. Available online at: https://www.hopkinsmedicine.org/health/conditions-and-diseases/head-injury (accessed October 12, 2023).

[B34] Hendrix N. Hendrix W. van Dijke K. Maresch B. Maas M. Bollen S.. (2023). Musculoskeletal radiologist-level performance by using deep learning for detection of scaphoid fractures on conventional multi-view radiographs of hand and wrist. Eur. Radiol. 33, 1575–1588. 10.1007/s00330-022-09205-4 36380195 PMC9935716

[B35] Henry C. Bowe C. O'Connell J. E. Ekanayake K. Kearns G. (2022). AB212. SOH22ABS193. Establishing a trauma database at the national maxillofacial unit. Mesentery Perit. 6, AB212. 10.21037/map-22-ab212

[B36] Hodson R. (2016). Precision medicine. Nature 537, S49. 10.1038/537S49a 27602738

[B37] Hooper T. Eccles G. Milliken T. Mathieu-Burry J. R. Reed W. (2019). Dose reduction in CT imaging for facial bone trauma in adults: a narrative literature review. J Med Radiat Sci. 66, 122–132. 10.1002/jmrs.319 30706691 PMC6545476

[B38] Hopper R. A. Salemy S. Sze R. W. (2006). Diagnosis of midface fractures with CT: what the surgeon needs to know. Radiographics 26, 783–793. 10.1148/rg.263045710 16702454

[B39] Hung K. Yeung A. W. K. Tanaka R. Bornstein M. M. (2020). Current applications, opportunities, and limitations of AI for 3D imaging in dental research and practice. Int. J. Environ. Res. Public Health 17, 4424. 10.3390/ijerph17124424 32575560 PMC7345758

[B40] Hung K. F. Yeung A. W. K. Bornstein M. M. Schwendicke F. (2023). Personalized dental medicine, artificial intelligence, and their relevance for dentomaxillofacial imaging. Dentomaxillofac. Radiol. 52, 20220335. 10.1259/dmfr.20220335 36472627 PMC9793453

[B41] Jeong T. S. Yee G. T. Kim K. G. Kim Y. J. Lee S. G. Kim W. K.. (2023). Automatically diagnosing skull fractures using an object detection method and deep learning algorithm in plain radiography images. J. Korean Neurosurg. Soc. 66, 53–62. 10.3340/jkns.2022.0062 35650677 PMC9837484

[B42] Jha D. Smedsrud P. H. Riegler M. A. Johansen D. de Lange T. Halvorsen P.. (2019). “ResUNet++: an advanced architecture for medical image segmentation,” in 2019 IEEE International Symposium on Multimedia (ISM) (San Diego, CA: IEEE), 225–2255. 10.1109/ISM46123.2019.00049

[B43] Jocher G. Chaurasia A. Stoken A. Borovec J. NanoCode012 Kwon Y. (2022). ultralytics/yolov5: v7.0 - *YOLOv5 SOTA realtime instance segmentation*. Zenodo. Available online at: 10.5281/zenodo.3908559 (accessed 05 April 2023).

[B44] Johnson K. B. Wei W. Q. Weeraratne D. Frisse M. E. Misulis K. Rhee K.. (2021). Precision medicine, AI, and the future of personalized health care. Clin. Transl. Sci. 14, 86–93. 10.1111/cts.12884 32961010 PMC7877825

[B45] Jones R. M. Sharma A. Hotchkiss R. Sperling J. W. Hamburger J. Ledig C.. (2020). Assessment of a deep-learning system for fracture detection in musculoskeletal radiographs. NPJ Digit. Med. 3, 144. 10.1038/s41746-020-00352-w 33145440 PMC7599208

[B46] Kalmet P. H. S. Sanduleanu S. Primakov S. Wu G. Jochems A. Refaee T.. (2020). Deep learning in fracture detection: a narrative review. Acta Orthop. 91, 215–220. 10.1080/17453674.2019.1711323 31928116 PMC7144272

[B47] Kaur J. Chopra R. (2010). Three dimensional CT reconstruction for the evaluation and surgical planning of mid face fractures: a 100 case study. J. Maxillofac. Oral Surg. 9, 323–328. 10.1007/s12663-010-0137-1 22190818 PMC3177467

[B48] Kaur N. Mishra G. Parihar V. Sharma S. S. Tyro K. D. Mann S. K.. (2023). Precision dentistry. Br. Dent. J. 234, 197. 10.1038/s41415-023-5586-2 36828996

[B49] Krogue J. D. Cheng K. V. Toogood K. M. Toogood P. Meinberg E. G. Geiger E. J.. (2020). Automatic hip fracture identification and functional subclassification with deep learning. Radiol. Artif. Intell. 2, 2. 10.1148/ryai.2020190023 33937815 PMC8017394

[B50] Kuang Z. Deng X. Yu L. Zhang H. Lin X. Ma H.. (2020). “Skull R-CNN: a CNN-based network for the skull fracture detection,” in Proceedings of the Third Conference on Medical Imaging with Deep Learning (Cambridge, MA: MIT Press), Vol. 121 (PMLR), 382–392.

[B51] Kuo R. Y. L. Harrison C. Curran T. A. Jones B. Freethy A. Cussons D.. (2022). Artificial intelligence in fracture detection: a systematic review and meta-analysis. Radiology 304, 211785. 10.1148/radiol.211785 35348381 PMC9270679

[B52] Langerhuizen D. W. G. Janssen S. J. Mallee W. H. van den Bekerom M. P. J. Ring D.. (2019). What are the applications and limitations of artificial intelligence for fracture detection and classification in orthopaedic trauma imaging? A systematic review. Clin. Orthop. Relat. Res. 477, 2482–2491. 10.1097/CORR.0000000000000848 31283727 PMC6903838

[B53] Lin T.-Y. Goyal P. Girshick R. He K. Dollár P. (2020). Focal loss for dense object detection. IEEE Trans. Pattern Anal. Mach. Intell. 42, 318–327. 10.1109/TPAMI.2018.2858826 30040631

[B54] Lind A. Akbarian E. Olsson S. Nasell H. Skoldenberg O. Razavian A. S.. (2021). Artificial intelligence for the classification of fractures around the knee in adults according to the 2018 AO/OTA classification system. PLoS ONE 16, e0248809. 10.1371/journal.pone.0248809 33793601 PMC8016258

[B55] Lindsey R. Daluiski A. Chopra S. Lachapelle A. Mozer M. Sicular S.. (2018). Deep neural network improves fracture detection by clinicians. Proc. Nat. Acad. Sci. 115, 11591–11596. 10.1073/pnas.1806905115 30348771 PMC6233134

[B56] Liu P. Lu L. Chen Y. Huo T. Xue M. Wang H.. (2022). Artificial intelligence to detect the femoral intertrochanteric fracture: the arrival of the intelligent-medicine era. Front Bioeng. Biotechnol. 10, 927926. 10.3389/fbioe.2022.927926 36147533 PMC9486191

[B57] Liu Y. Liu G. Zhang Q. (2019). Deep learning and medical diagnosis. Lancet 394, 1709–1710. 10.1016/S0140-6736(19)32501-2 31709993

[B58] Liu Y. Liu W. Chen H. Xie S. Wang C. Liang T.. (2023). Artificial intelligence versus radiologist in the accuracy of fracture detection based on computed tomography images: a multi-dimensional, multi-region analysis. Quant. Imaging Med. Surg. 13, 10. 10.21037/qims-23-428 37869340 PMC10585498

[B59] Liu Z. Lin Y. Cao Y. Hu H. Wei Y. Zhang Z.. (2021). “Swin transformer: hierarchical vision transformer using shifted windows,” in Proceedings of the IEEE/CVF International Conference on Computer Vision (Montreal, QC: IEEE), 10012–10022. 10.1109/ICCV48922.2021.00986

[B60] Ludi E. K. Rohatgi S. Zygmont M. E. Khosa F. Hanna T. N. (2016). Do radiologists and surgeons speak the same language? A retrospective review of facial trauma. Am. J. Roentgenol. 207, 1070–1076. 10.2214/AJR.15.15901 27556232

[B61] Markus A. F. Kors J. A. Rijnbeek P. R. (2021). The role of explainability in creating trustworthy artificial intelligence for health care: a comprehensive survey of the terminology, design choices, and evaluation strategies. J. Biomed. Inform. 113, 103655. 10.1016/j.jbi.2020.103655 33309898

[B62] Meena T. Roy S. (2022). Bone fracture detection using deep supervised learning from radiological images: a paradigm shift. Diagnostics 12, 2420. 10.3390/diagnostics12102420 36292109 PMC9600559

[B63] Mistry R. K. Hohman M. H. Al-Sayed A. A. (2023). Facial nerve trauma. Treasure Island, FL: StatPearls Publishing. Available online at: https://www.ncbi.nlm.nih.gov/books/NBK553095/ (accessed March 30, 2023). 31971735

[B64] Moawad A. W. Fuentes D. T. ElBanan M. G. Shalaby A. S. Guccione J. Kamel S.. (2022). Artificial intelligence in diagnostic radiology: where do we stand, challenges, and opportunities. J. Comput. Assist. Tomogr. 46, 78–90. 10.1097/RCT.0000000000001247 35027520

[B65] Moon G. Kim S. Kim W. Kim Y. Jeong Y. Choi H. S.. (2022). Computer aided facial bone fracture diagnosis (CA-FBFD) system based on object detection model. IEEE Access 10, 79061–79070. 10.1109/ACCESS.2022.3192389

[B66] Murphy E. A. Ehrhardt B. Gregson C. L. von Arx O. A. Hartley A. Whitehouse M. R.. (2022). Machine learning outperforms clinical experts in classification of hip fractures. Sci. Rep. 12, 2058. 10.1038/s41598-022-06018-9 35136091 PMC8825848

[B67] Nguyen T. Maarek R. Hermann A. L. Kammoun A. Marchi A.. (2022). Assessment of an artificial intelligence aid for the detection of appendicular skeletal fractures in children and young adults by senior and junior radiologists. Pediatr. Radiol. 52, 2215–2226. 10.1007/s00247-022-05496-3 36169667

[B68] Norori N. Hu Q. Aellen F. M. Faraci F. D. Tzovara A. (2021). Addressing bias in big data and AI for health care: a call for open science. Patterns 2, 100347. 10.1016/j.patter.2021.100347 34693373 PMC8515002

[B69] Nowak A. J. (2021). “Artificial intelligence in evidence-based medicine,” in Artificial Intelligence in Medicine, eds N. Lidstromer, and H. Ashrafian (Cham: Springer) 12. 10.1007/978-3-030-58080-3_43-1

[B70] Oakden-Rayner L. Gale W. Bonham T. A. Lungren M. P. Carneiro G. Bradley A. P.. (2022). Validation and algorithmic audit of a deep learning system for the detection of proximal femoral fractures in patients in the emergency department: a diagnostic accuracy study. Lancet Digit. Health 4, e351–e358. 10.1016/S2589-7500(22)00004-8 35396184

[B71] Oppenheimer J. Luken S. Hamm B. Niehues S. M. (2023). A prospective approach to integration of AI fracture detection software in radiographs into clinical workflow. Life 13, 223. 10.3390/life13010223 36676172 PMC9864518

[B72] Ozturk O. Kutucu H. (2017). “Detection of bone fractures using image processing techniques and artificial neural networks,” in 2017 International Artificial Intelligence and Data Processing Symposium (IDAP) (Malatya: IEEE), 1–5. 10.1109/IDAP.2017.8090311

[B73] Parpaleix A. Parsy C. Cordari M. Mejdoubi M. (2023). Assessment of a combined musculoskeletal and chest deep learning-based detection solution in an emergency setting. Eur. J. Radiol. Open 10, 100482. 10.1016/j.ejro.2023.100482 36941993 PMC10023863

[B74] Patel B. C. Wright T. Waseem M. (2022). Le Fort fractures. Treasure Island, FL: StatPearls Publishing. Available online at: https://www.ncbi.nlm.nih.gov/books/NBK526060/ (accessed March 30, 2023). 30252316

[B75] Pham T. D. Ravi V. Fan C. W. Luo B. Sun X. F. (2023a). Classification of IHC images of NATs with deep learning and fuzzy recurrence plots for predicting survival rates of rectal-cancer patients. IEEE J. Transl. Eng. Health Med. 11, 87–95. 10.1109/JTEHM.2022.3229561 36704244 PMC9870269

[B76] Pham T. D. Ravi V. Luo B. Fan C. Sun X. F. (2023b). Artificial intelligence fusion for predicting survival of rectal cancer patients using immunohistochemical expression of Ras homolog family member B in biopsy. Explor. Target. Anti-Tumor ther. 4, 889–904. 10.37349/etat.2023.00119 36937315 PMC10017185

[B77] Pham T. D. Sun X. F. (2023). “Wavelet scattering of RhoB-expressed deep-learning features for rectal cancer prognosis,” *2023 IEEE International Symposium on Biomedical Imaging* (ISBI 2023, 18–21 April 2023, Cartagena de Indias, Colombia). 10.1109/ISBI53787.2023.10230417

[B78] Pinto A. Brunese L. (2010). Spectrum of diagnostic errors in radiology. World J. Radiol. 2, 377–383. 10.4329/wjr.v2.i10.377 21161023 PMC2999012

[B79] Pinto A. Berritto D. Russo A. Riccitiello F. Caruso M. Belfiore M. P.. (2018). Traumatic fractures in adults: missed diagnosis on plain radiographs in the emergency department. Acta Biomed. 89, 111–123. 10.23750/abm.v89i1-S.7015 29350641 PMC6179080

[B80] Piraianu A.-I. Fulga A. Musat C. L. Ciobotaru O.-R. Poalelungi D. G. Stamate E.. (2023). Enhancing the evidence with algorithms: how artificial intelligence is transforming forensic medicine. Diagnostics 13, 2992. 10.3390/diagnostics13182992 37761359 PMC10529115

[B81] Rainey C. McConnell J. Hughes C. Bond R. McFadden S. (2021). Artificial intelligence for diagnosis of fractures on plain radiographs: a scoping review of current literature. Intell.-Based Med. 5, 100033. 10.1016/j.ibmed.2021.100033

[B82] Ramgopal S. Sanchez-Pinto L. N. Horvat C. M. Carroll M. S. Luo Y. Florin T. A.. (2023). Artificial intelligence-based clinical decision support in pediatrics. Pediatr. Res. 93, 334–341. 10.1038/s41390-022-02226-1 35906317 PMC9668209

[B83] Redmon J. Divvala S. Girshick R. Farhadi A. (2015). You only look once: unified, real-time object detection. arXiv [preprint]. 10.48550/arXiv.1506.02640

[B84] Ren M. Yi P. H. (2021). Deep learning detection of subtle fractures using staged algorithms to mimic radiologist search pattern. Skeletal Radiol. 51, 345–353. 10.1007/s00256-021-03739-2 33576861

[B85] Ren S. Q. He K. M. Girshick R. Sun J. (2017). Faster R-CNN: towards real-time object detection with region proposal networks. IEEE Trans. Pattern Anal. Mach. Intell. 39, 1137–1149. 10.1109/TPAMI.2016.2577031 27295650

[B86] Ronneberger O. Fischer P. Brox T. (2015). “U-net: convolutional networks for biomedical image segmentation,” in Medical Image Computing and Computer-Assisted Intervention (MICCAI 2015). Lecture Notes in Computer Science, eds N. Navab, J. Hornegger, W. Wells, and A. Frangi (Cham: Springer), 9351. 10.1007/978-3-319-24574-4_28

[B87] Rosello E. G. Granado A. M. Q. Garcia M. A. Martí S. J. Sala G. L. Marmol B. B.. (2020). Facial fractures: classification and highlights for a useful report. Insights Imaging 11, 49. 10.1186/s13244-020-00847-w 32193796 PMC7082488

[B88] Rubin D. L. (2019). Artificial intelligence in imaging: the radiologist's role. J. Am. Coll. Radiol. 16, 1309–1317. 10.1016/j.jacr.2019.05.036 31492409 PMC6733578

[B89] Ryu J. Y. Chung H. Y. Choi K. Y. (2021). Potential role of artificial intelligence in craniofacial surgery. Arch. Craniofac. Surg. 22, 223–231. 10.7181/acfs.2021.00507 34732033 PMC8568494

[B90] Sackett D. L. Rosenberg W. M. Gray J. A. Haynes R. B. Richardson W. S. (1996). Evidence based medicine: what it is and what it isn't. BMJ 312, 71–72. 10.1136/bmj.312.7023.71 8555924 PMC2349778

[B91] Sato Y. Takegami Y. Asamoto T. Ono Y. Hidetoshi T. Goto R.. (2021). Artificial intelligence improves the accuracy of residents in the diagnosis of hip fractures: a multicenter study. BMC Musculoskelet. Disord. 22, 407. 10.1186/s12891-021-04260-2 33941145 PMC8091525

[B92] Scarfe W. C. (2005). Imaging of maxillofacial trauma: evolutions and emerging revolutions. Oral Surgery, Oral Medicine, Oral Pathology, Oral Radiology, and Endodontology 100, S75–S96. 10.1016/j.tripleo.2005.05.057 16037795

[B93] Schwendicke F. Krois J. (2022). Precision dentistry–what it is, where it fails (yet), and how to get there. Clin. Oral. Invest. 26, 3395–3403. 10.1007/s00784-022-04420-1 PMC8918420 35284954

[B94] Seol Y. J. Kim Y. J. Kim Y. S. Cheon Y. W. Kim K. G. (2022). A study on 3D deep learning-based automatic diagnosis of nasal fractures. Sensors 22, 506. 10.3390/s22020506 35062465 PMC8780993

[B95] Shah S. Uppal S. K. Mittal R. K. Garg R. Saggar K. Dhawan R.. (2016). Diagnostic tools in maxillofacial fractures: as there really a need of three-dimensional computed tomography? Indian J. Plast. Surg. 49, 225–233. 10.4103/0970-0358.191320 27833286 PMC5052996

[B96] Shan W. Guo J. Mao X. Zhang Y. Huang Y. Wang S.. (2021). Automated identification of skull fractures with deep learning: a comparison between object detection and segmentation approach. Front. Neurol. 12, 687931. 10.3389/fneur.2021.687931 34777193 PMC8585755

[B97] Shelmerdine S. C. White R. D. Liu H. Arthurs O. J. Sebire N. J. (2022). Artificial intelligence for radiological paediatric fracture assessment: a systematic review. Insights Imaging 13, 94. 10.1186/s13244-022-01234-3 35657439 PMC9166920

[B98] Simonyan K. Zisserman A. (2014). “Two-stream convolutional networks for action recognition in videos,” in Proceedings of the 27th International Conference on Neural Information Processing Systems (NIPS'14)-1 (Cambridge, MA: MIT Press), 568–576.

[B99] Son D. M. Yoon Y. A. Kwon H. J. Lee S. H. (2022). Combined deep learning techniques for mandibular fracture diagnosis assistance. Life 12, 1711. 10.3390/life12111711 36362866 PMC9697461

[B100] Tsopra R. Fernandez X. Luchinat C. Alberghina L. Lehrach H. Vanoni M.. (2021). A framework for validating AI in precision medicine: considerations from the European ITFoC consortium. BMC Med. Inform. Decis. Mak. 21, 274. 10.1186/s12911-021-01634-3 34600518 PMC8487519

[B101] Uddin M. Wang Y. Woodbury-Smith M. (2019). Artificial intelligence for precision medicine in neurodevelopmental disorders. NPJ Digit. Med. 2, 112. 10.1038/s41746-019-0191-0 31799421 PMC6872596

[B102] Vasconcelos H. Jorke M. Grunde-McLaughlin M. Gerstenberg T. Bernstein M. Krishna R. (2023). Explanations can reduce overreliance on AI systems during decision-making. arXiv [preprint]. 10.48550/arXiv.2212.06823

[B103] Vinayahalingam S. van Nistelrooij N. van Ginneken B. Bressem K. Tröltzsch D. Heiland M.. (2022). Detection of mandibular fractures on panoramic radiographs using deep learning. Sci. Rep. 12, 19596. 10.1038/s41598-022-23445-w 36379971 PMC9666517

[B104] Vujcich N. Gebauer D. (2018). Current and evolving trends in the management of facial fractures. Aust. Dent. J. 63(S1), S35–S47. 10.1111/adj.12589 29574816

[B105] Wang H. C. Wang S. C. Yan J. L. Ko L. W. (2023). Artificial Intelligence model trained with sparse data to detect facial and cranial bone fractures from head CT. J. Digit. Imaging 36, 1408–1418. 10.1007/s10278-023-00829-6 37095310 PMC10407005

[B106] Wang X. Xu Z. Tong Y. Xia L. Jie B. Ding P.. (2022). Detection and classification of mandibular fracture on CT scan using deep convolutional neural network. Clin. Oral Investig. 26, 4593–4601. 10.1007/s00784-022-04427-8 35218428

[B107] Warin K. Limprasert W. Suebnukarn S. Paipongna T. Jantana P. Vicharueang S.. (2023). Maxillofacial fracture detection and classification in computed tomography images using convolutional neural network-based models. Sci. Rep. 13, 3434. 10.1038/s41598-023-30640-w 36859660 PMC9978019

[B108] Wilson J. Daugherty P. R. (2018). “Collaborative intelligence: humans and AI are joining forces,” in Harvard Business Review July-August 2018 (Cambridge, MA: MIT Press), 114–123.

[B109] Wu J. Liu N. Li X. Fan Q. Li Z. Shang J.. (2013). Convolutional neural network for detecting rib fractures on chest radiographs: a feasibility study. BMC Med. Imaging 23, 18. 10.1186/s12880-023-00975-x 36717773 PMC9885575

[B110] Yang C. Yang L. Gao G. D. Zong H. Q. Gao D. (2022). Assessment of artificial intelligence-aided reading in the detection of nasal bone fractures. Technol. Health Care 31, 1017–1025. 10.3233/THC-220501 36442167

[B111] Yoon A. P. Lee Y. Kane R. L. Kuo C. Lin C. Chung K. C.. (2021). Development and validation of a deep learning model using convolutional neural networks to identify scaphoid fractures in radiographs. JAMA Netw. Open 4, e216096. 10.1001/jamanetworkopen.2021.6096 33956133 PMC8103226

[B112] Zech J. R. Santomartino S. M. Yi P. H. (2022). Artificial intelligence (AI) for fracture diagnosis: an overview of current products and considerations for clinical adoption, from the AJR Special Series on AI Applications. Am. J. Roentgenol. 219, 869–878. 10.2214/AJR.22.27873 35731103

